# Telomeric Repeats Facilitate CENP-A^Cnp1^ Incorporation via Telomere Binding Proteins

**DOI:** 10.1371/journal.pone.0069673

**Published:** 2013-07-31

**Authors:** Araceli G. Castillo, Alison L. Pidoux, Sandra Catania, Mickaël Durand-Dubief, Eun Shik Choi, Georgina Hamilton, Karl Ekwall, Robin C. Allshire

**Affiliations:** 1 Wellcome Trust Centre for Cell Biology and Institute of Cell Biology, School of Biological Sciences, the University of Edinburgh, Edinburgh, Scotland, United Kingdom; 2 Department of Biosciences and Nutrition, Karolinska Institutet, NOVUM, Huddinge, Sweden; Duke University, United States of America

## Abstract

The histone H3 variant, CENP-A, is normally assembled upon canonical centromeric sequences, but there is no apparent obligate coupling of sequence and assembly, suggesting that centromere location can be epigenetically determined. To explore the tolerances and constraints on CENP-A deposition we investigated whether certain locations are favoured when additional CENP-A^Cnp1^ is present in fission yeast cells. Our analyses show that additional CENP-A^Cnp1^ accumulates within and close to heterochromatic centromeric outer repeats, and over regions adjacent to rDNA and telomeres. The use of minichromosome derivatives with unique DNA sequences internal to chromosome ends shows that telomeres are sufficient to direct CENP-A^Cnp1^ deposition. However, chromosome ends are not required as CENP-A^Cnp1^ deposition also occurs at telomere repeats inserted at an internal locus and correlates with the presence of H3K9 methylation near these repeats. The Ccq1 protein, which is known to bind telomere repeats and recruit telomerase, was found to be required to induce H3K9 methylation and thus promote the incorporation of CENP-A^Cnp1^ near telomere repeats. These analyses demonstrate that at non-centromeric chromosomal locations the presence of heterochromatin influences the sites at which CENP-A is incorporated into chromatin and, thus, potentially the location of centromeres.

## Introduction

Centromeres are the chromosomal locations at which kinetochores, the machinery responsible for accurate chromosome segregation, are assembled. Kinetochores are assembled on unusual chromatin composed of nucleosomes in which canonical histone H3 is replaced by a centromere specific histone H3 variant, generally known as CENP-A. The incorporation of CENP-A is critical for providing the foundation for the assembly of kinetochores and thus specifying the location of active centromeres in most eukaryotes. Kinetochores are normally assembled on specific sequences such as centromeric repeat sequences (α-satellite in human) and these DNA sequences can direct the *de novo* assembly of functional kinetochores. Such analyses indicate that specific DNA sequences can direct events that promote CENP-A incorporation and kinetochore assembly. Although kinetochores are normally assembled upon these preferred, defined centromeric sequences in any particular organism, there is now an abundance of examples demonstrating that centromeric sequences are sometimes not sufficient and sometimes not necessary for kinetochore assembly. On human dicentric chromosomes, one centromere can be inactivated despite the continued presence of α-satellite sequence at both centromeric loci [1,2]. However, other observations indicate that specific DNA sequences are not required and that the location of CENP-A incorporation and kinetochore assembly is epigenetically regulated. For example, chromosome rearrangements that delete the normal centromere can allow kinetochores to assemble at novel locations, known as neocentromeres. At such neocentromeres, the underlying DNA sequence can be non-repetitive and bears no sequence similarity to the DNA elements associated with normal centromeres. Neocentromeres have been observed and characterised in a variety of organisms including humans, flies, yeasts and plants [3–10]. Moreover, in several species natural centromeres have been identified which are located at non-repetitive unique DNA sequences [11]. These centromeres are thought to represent recently established centromeres and presumably arise through the mechanisms that also lead to neocentromere formation.

Together such observations suggest that centromeres are normally assembled upon canonical centromeric sequences in any particular species, but there is no obligate coupling of sequence and assembly. These observations provide strong evidence for an epigenetic component to the regulation of centromere assembly. Several lines of evidence point to the histone H3 variant, CENP-A being the epigenetic mark that specifies centromere identity. It is found only at active centromeres, including neocentromeres and is absent from inactivated centromeres [1]. Tethering of CENP-A^CID^ in *Drosophila* promotes incorporation of endogenous CENP-A^CID^, even after the tethered version is removed, suggesting that CENP-A chromatin is able to direct its own propagation [12]. This is also supported by experiments in human cells in which tethering of the CENP-A chaperone HJURP to LacO arrays led to CENP-A incorporation and kinetochore assembly at a non centromeric location [13].

The incorporation of CENP-A at novel sites could be influenced in various ways. CENP-A may be rapidly turned over to prevent its accumulation at non-centromeric locations [14,15]. Active processes such as transcription can promote H3 but block CENP-A incorporation [16]. However, particular chromatin features may provide an environment that favours the incorporation of CENP-A and its stabilisation in chromatin relative to canonical histone H3. Indeed the centromeric DNA elements on which CENP-A is naturally assembled, have been shown to be transcribed in a variety of organisms [17].

The three centromeres on fission yeast (*Schizosaccharomyces pombe*) chromosomes are composed of a central domain (*cnt* plus *imr*) flanked by repetitive DNA elements known as the outer repeats (*otr*). CENP-A^Cnp1^ chromatin and the kinetochore are assembled over the central domain (~10 kb) while heterochromatin, in which histone H3 is methylated on lysine 9 (H3K9me) that allows the binding of specific chromodomain proteins, coats the outer repeats. Central domain DNA alone is unable to direct the de novo assembly of CENP-A^Cnp1^ chromatin on this DNA following its introduction into cells. The flanking heterochromatin is required to provide an environment that somehow allows the replacement of histone H3 with CENP-A^Cnp1^ and kinetochore assembly over the nearby central domain DNA [18,19]. CENP-A^Cnp1^ might be attracted to non-centromeric sites by sequences possessing certain features, but also by chromatin context. The importance of chromatin context is demonstrated by the fact that heterochromatin is required for the establishment of CENP-A^Cnp1^ chromatin on adjacent central core sequences, but once established this heterochromatin is not required for the maintenance of CENP-A^Cnp1^ [18,19].

Apart from centromeres a domain of heterochromatin is formed over the 15 kb region encompassing the silent mating type loci and an extended region internal to telomeres. Heterochromatin is not essential in fission yeast and its formation at all locations is dependent on a single gene encoding the H3 K9 methyltransferase, Clr4. Non-coding transcripts that emanate from repetitive elements at centromeres, telomeres and the mating type locus, are processed to small interfering RNAs which direct H3K9 methylation by Clr4. This modification creates a binding site for the chromodomain protein Swi6. Histones H3 and H4 in the nucleosomes of outer repeat chromatin are underacetylated on lysines in their N-terminal tails and this hypoacetylated state results form the recruitment of the histone deacetylases: Clr3, Clr6 and Sir2 [20].

The engineered deletion of a fission yeast centromere (*cen1*) has been shown to allow neocentromeres to form at other chromosomal locations. At these neocentromeres CENP-A^Cnp1^ is associated with DNA sequences that lack homology to the central domain of centromeres, where CENP-A^Cnp1^ is normally incorporated. All neocentromeres formed were within 100 kb of either the left or right telomeres. Heterochromatin is associated with fission yeast telomeres and in the absence of heterochromatin the frequency of neocentromere formation was reduced [6].

Fission yeast telomeres are composed of ~300 bp of terminal GT rich repeats that are added by telomerase, internal to these are telomere associated sequences (TAS) on chromosome 1 and 2 [21]. These TAS repeat elements share a short region of homology with centromeric outer repeats [22,23]. The terminal TTAC(A) GG(G_1-4_) repeats are bound by specific proteins that form a specialised structure that regulates telomerase recruitment, protects chromosomal ends from degradation and anchors them at the nuclear periphery [24]. Telomere repeat length is regulated by telomere associated proteins that are recruited by Taz1, which directly binds double stranded (ds) repeats [25], and Pot1 which binds the single stranded (ss) overhang at the extreme end [26]. Taz1 recruits Rap1 and Rif1 to double stranded telomeric DNA [27,28], whereas Pot1 recruits Tpz1 to the single stranded G tail [29]. In complex with two other factors, Ccq1 (which interacts with Pot1-Tpz1 alone) and Poz1 (which bridges the ss and ds regions of telomeres through direct interactions with Pot1-Tpz1 as well as Rap1), these proteins regulate telomere length and protect the ends of chromosomes [29]. Ccq1 also associates with the SHREC/Clr3 histone deacetylase complex and recruits it to telomeres where it participates in silencing [30].

Taz1 has been shown to inhibit telomerase-mediated telomere elongation, by ensuring cell cycle dependent telomerase recruitment to telomeres [31,32]. Rap1 and Rif1, which associate with Taz1, have also been found to negatively regulate telomere length, both in a Taz1 dependent and independent manner [27,28,33]. Conversely, Pot1 and its associated factors Tpz1 and Ccq1 have been shown to promote telomere elongation and are required for stable telomere maintenance [26,29,34]. In particular, Ccq1 has also been shown to directly interact with telomerase and recruit it to telomeres [35].

To explore the tolerances and constraints on CENP-A^Cnp1^ deposition we investigated whether certain locations are favoured by CENP-A^Cnp1^ when present in excess. We find that when CENP-A^Cnp1^ is moderately overexpressed in fission yeast it accumulates in telomeric regions, the same regions that can form neocentromeres. Here we use engineered telomeres on minichromosomes, integrated arrays of telomeric repeats and cells lacking telomere and heterochromatin proteins to investigate what features promote CENP-A^Cnp1^ incorporation in the telomeric regions of chromosomes. Our analyses show that a short array of telomere repeats, independent of a chromosome end, is sufficient to promote CENP-A^Cnp1^ incorporation and that Ccq1 plays a specific role in promoting the formation of nearby heterochromatin and CENP-A^Cnp1^ incorporation.

## Results

### Additional CENP-A^Cnp1^ accumulates at ectopic sites

CENP-A^Cnp1^ is normally restricted to the central domain of fission yeast centromeres. However, our previous analyses show that CENP-A^Cnp1^ is able to assemble on non-centromeric DNA inserted within the central domain [36]. Moreover, moderate overexpression of CENP-A^Cnp1^ causes increased repression of a marker gene inserted in the central core and increased levels of CENP-A^Cnp1^ on that marker gene. It has also been shown that the deletion of an endogenous centromere (*cen1*) results in CENP-A^Cnp1^ accumulating over an extended euchromatic region adjacent to telomeres, forming neocentromeres [6]. These observations suggest that although CENP-A^Cnp1^ is normally restricted to the central domain at centromeres, it can assemble upon non-centromeric sequences.

**Figure 1:**
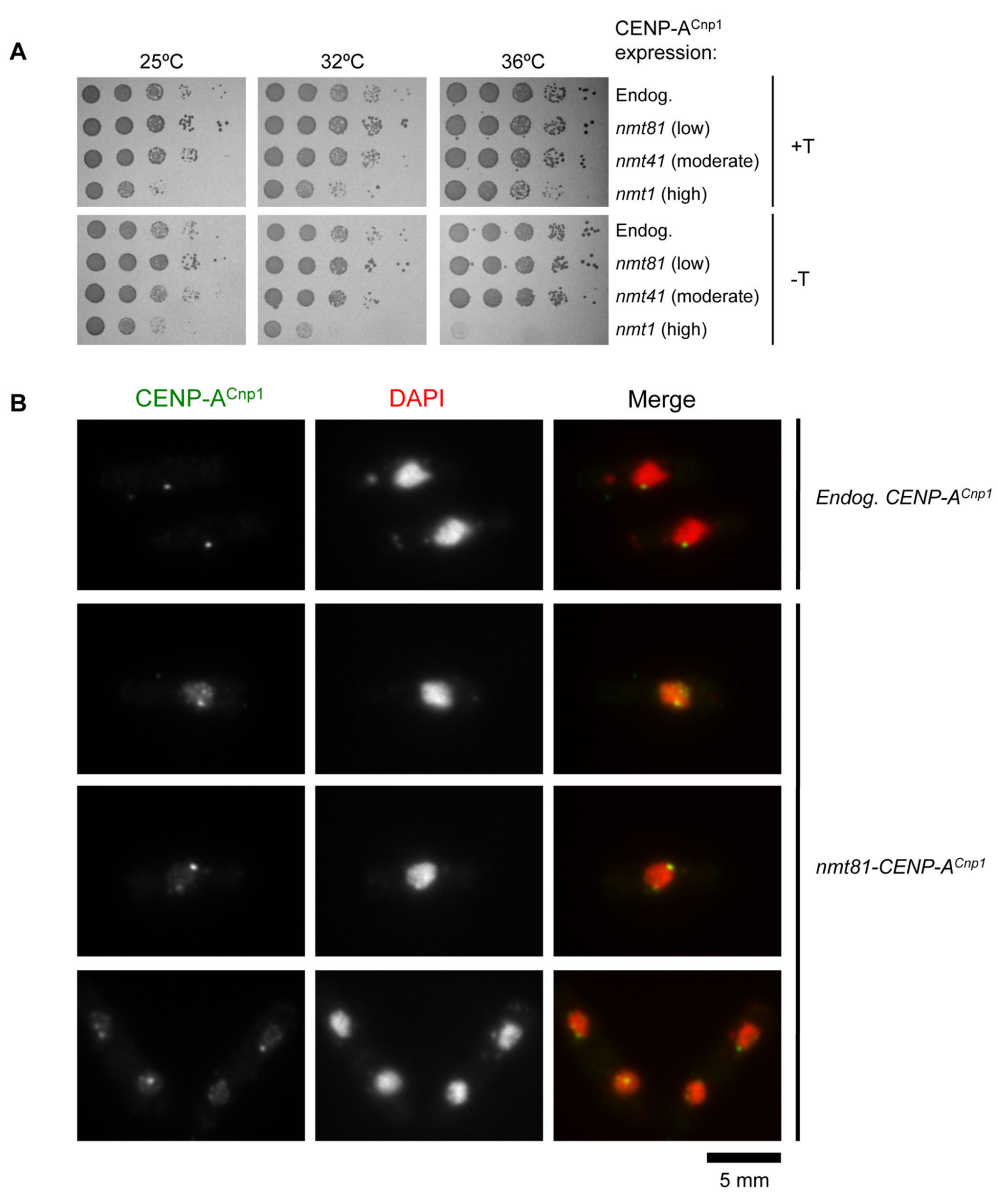
Additional CENP-A^Cnp1^ accumulates at discrete foci in *S. pombe* cells. CENP-A^Cnp1^ was expressed from different strengths of the thiamine repressible *nmt* promoter (*nmt81* – weak, *nmt41* – medium, *nmt1* – strong); integrated at *ars1* in chromosome 1. A strain that has an empty *nmt* promoter plasmid integrated was used as a control (Endog: CENP-A^Cnp1^ endogenous levels). (**A**) Serial dilution assays to monitor the effect of additional CENP-A^Cnp1^ on cell growth. Cells were spotted on minimal media with (+T) or without (-T) thiamine at three different temperatures, 25^°^C, 32^°^C and 36^°^C. (**B**) Immunolocalization of CENP-A^Cnp1^ in cells expressing endogenous levels of CENP-A^Cnp1^ or extra CENP-A^Cnp1^ expressed from the *nmt81* promoter. Cells were grown in the absence of thiamine for 24 h at 36^o^C and stained with anti-CENP-A^Cnp1^ antibody (green) and DAPI (DNA: red). Scale bar, 5 µm.

In order to further investigate the constraints or promiscuity of CENP-A^Cnp1^ deposition we expressed additional CENP-A^Cnp1^ at a variety of levels and conditions. The CENP-A^Cnp1^ open reading frame was expressed from different strengths of the thiamine repressible *nmt* promoter (*nmt81* – weak, *nmt41* – medium, *nmt1* – strong) [37], integrated at the same chromosomal location (this work and [16,36], Figures S1A and S1B). The effect on cell growth and viability was assessed at different temperatures in serial dilution assays (Figure 1A). Whilst expression of moderate or medium additional amounts of CENP-A^Cnp1^ was tolerated, strong overexpression had a highly deleterious effect on cell growth, especially at 36^o^C.

To determine whether the observed effect on growth was accompanied by deposition of CENP-A^Cnp1^ at non-centromeric loci, CENP-A^Cnp1^ localisation was examined in cells expressing additional CENP-A^Cnp1^ from the three types of promoters. During interphase, the three fission yeast centromeres are clustered adjacent to the spindle pole body (SPB; centrosome equivalent) and appear as a single dot (Figure 1B). When overexpressed at low levels, although the signal intensity was increased, a single CENP-A^Cnp1^ dot was visible at 25^o^C. In contrast, at 36^o^C additional CENP-A^Cnp1^ foci were observed that were distinct from the main centromeric cluster (Figure 1B). The most common pattern observed was a few (2–6) bright foci of staining in addition to the centromeric spot. In addition, a general increase of staining of all chromatin, but not the entire nuclear volume, was also observed under moderate or high expression conditions (Figure S1C), suggesting that CENP-A^Cnp1^ was incorporated to some extent more broadly throughout chromatin. These observations indicate that when CENP-A^Cnp1^ is overexpressed it can be deposited at chromatin contexts distinct from centromeres and this is dependent on the level of expression.

### Additional CENP-A^Cnp1^ accumulates on chromatin at pericentromeres, and adjacent to telomeres and rDNA

**Figure 2:**
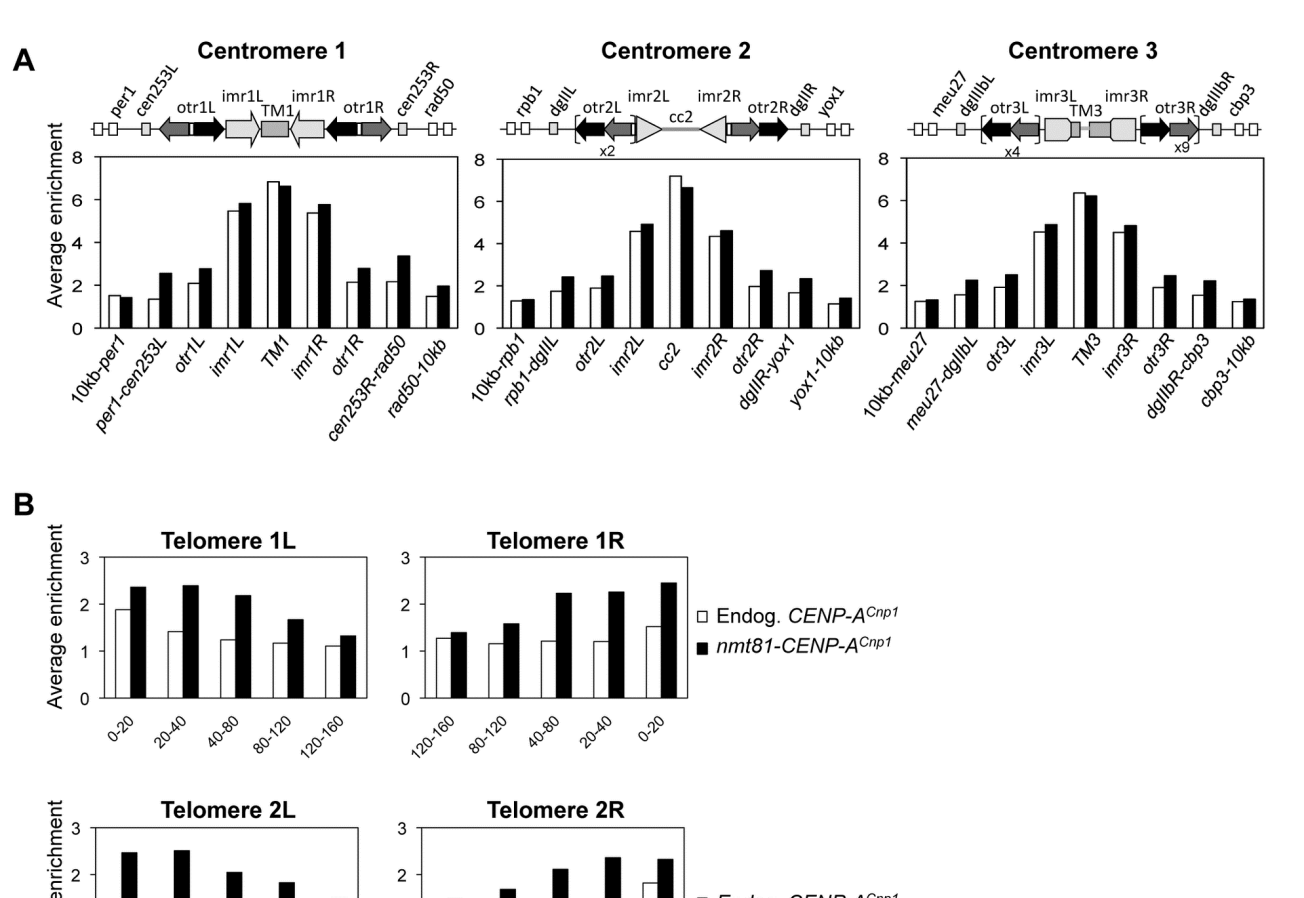
Additional CENP-A^Cnp1^ is deposited at sub-telomeric and pericentromeric regions. ChIP-chip analysis of CENP-A^Cnp1^ levels at cells with moderate expression of additional CENP-A^Cnp1^ (*nmt81*-CENP-A^Cnp1^) or wild-type protein levels (Endog. CENP-A^Cnp1^). (**A**) Average enrichment of CENP-A^Cnp1^ over different chromosomal regions (*cnt*, *imr*, *otr*) of centromeres I, II and III (X-axis, linear scale). The number of repetitive elements at different loci is indicated (x2, x4, x9). The average enrichment over 10 kb regions immediately adjacent to the centromeric *otr* repeats is also shown (10 kb region encompassing the most proximal annotated gene on each side of each centromere; *per1* and *rad50* for *cen1*, *rpb1* and *yox1* for *cen2* and *meu27* and *cbp3* for *cen3*). (**B**) Average enrichment from ChIP-chip analyses of CENP-A^Cnp1^ over 120 kb regions from the left or right telomere of each chromosome. Signal intensity was averaged within 20 kb or 40 kb windows as indicated. Left panels, left telomeres; right panels, right telomeres of each chromosome.

To accurately determine the location of CENP-A^Cnp1^ in cells that display strong extra foci with low background chromatin signal, anti-CENP-A^Cnp1^ genome-wide chromatin immunoprecipitation (ChIP-chip) analysis was performed on cells with low CENP-A^Cnp1^ overexpression (*nmt81-CENP-A* ^*Cnp1*^) and cells with wild-type CENP-A^Cnp1^ levels (36^o^C; see Materials and Methods and Figure 1B). As expected, CENP-A^Cnp1^ was observed at the central domains of all three fission yeast centromeres (Figure 2A) in both cell types.

**Figure 3:**
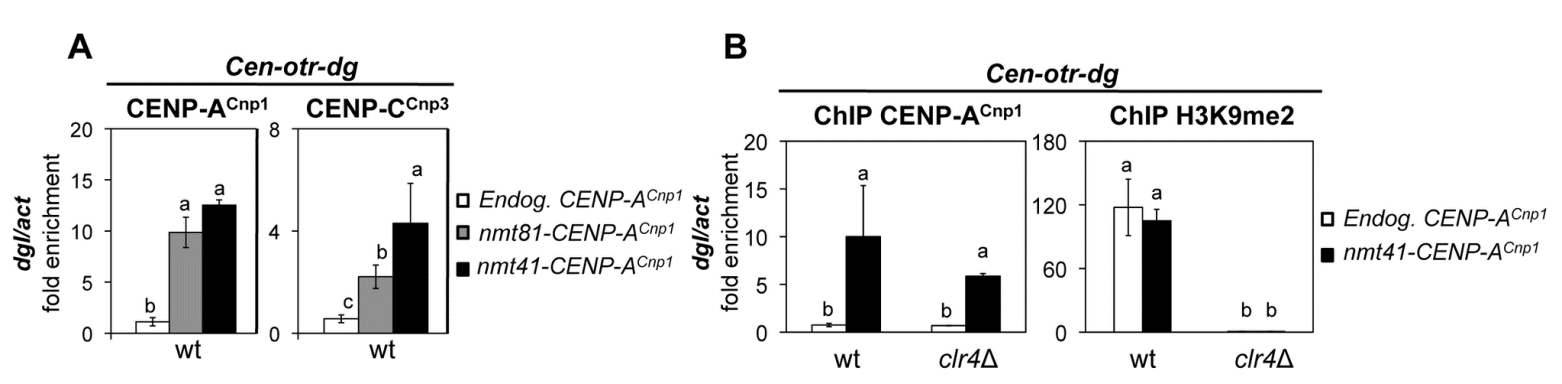
CENP-A^Cnp1^ can accumulate on centromeric repeat regions independently of H3K9 methylation. (**A**) ChIP-qPCR-analysis of CENP-A^Cnp1^ and CENP-C^Cnp3^ levels on centromeric outer repeat sequences (*otr dgI*) in cells expressing endogenous (Endog. CENP-A^Cnp1^) or additional (*nmt81*-CENP-A^Cnp1^ or *nmt41*-CENP-A^Cnp1^) levels of CENP-A^Cnp1^. (**B**) ChIP-qPCR analysis of CENP-A^Cnp1^ and H3K9me2 levels on centromeric *otr dgI* repeats in wild-type and *clr4*∆ cells expressing endogenous (Endog. CENP-A^Cnp1^) or additional levels (*nmt41*-CENP-A^Cnp1^) of CENP-A^Cnp1^. Enrichment on *dgI* was normalized to the signal obtained for the euchromatic gene encoding actin (*act1* ^+^). Error bars indicate S.D. from 3 biological replicates. Mean values marked with different letter (a, b or c) indicate results significantly different from each other, as established by One Way ANOVA and Holm-Sidak test for multiple comparison (P<0.01).

CENP-A^Cnp1^ is normally restricted to the central domain of centromeres and does not occupy the heterochromatic centromere outer repeats [36,38]. Examination of the ChIP-chip data indicates that additional CENP-A^Cnp1^ accumulated on the outer repeats and pericentromere regions (Figure 2A and Figure S2A), and this was verified by qPCR and duplex PCR using cells expressing low (*nmt81*) or moderate (*nmt41*) levels of CENP-A^Cnp1^ (Figure 3A Figure S2B). Interestingly, in cells expressing additional CENP-A^Cnp1^, the kinetochore protein CENP-C^Cnp3^ was also detected on the centromeric outer repeats (Figure 3A). Normally the outer repeats are packaged in chromatin marked by H3K9 methylation which is dependent on the Clr4 histone methyltransferase [20,39] (Figure 3B). Additional CENP-A^Cnp1^ is still detected over the centromere outer repeats in a *clr4Δ* mutant (Figure 3B). These analyses suggest that additional CENP-A^Cnp1^ can be attracted to centromeric regions by the presence of CENP-A^Cnp1^ and associated kinetochore proteins within the central domain.

Strikingly, in cells expressing additional CENP-A^Cnp1^ the levels of CENP-A^Cnp1^ are elevated over an extended region adjacent to the left and right telomeres of chromosomes 1 and 2 (Figure 2B and Figure S2A). These subtelomeric domains of CENP-A^Cnp1^ enrichment encompassed ~100 kb in a series of discrete peaks that were mainly located at intergenic regions. Four *S. pombe* telomeres (chromosomes 1 and 2) have telomere-associated sequences (TAS) internal to the TTAC(A) GG(G_1-4_) repeats [21]. Arrays of rDNA directly abut the telomeres of chromosome 3 and increased CENP-A^Cnp1^ levels were observed on the unique DNA immediately internal to the rDNA (Figure 2B and Figure S2A). This increased incorporation of CENP-A^Cnp1^ at subtelomeric regions and adjacent to rDNA was verified by duplex PCR (Figure S2C) and qPCR (Figure S2D).

These analyses show that when expressed in excess, the normally exclusively centromeric histone CENP-A^Cnp1^ is deposited at additional loci including centromeric outer repeats / pericentromeric regions, adjacent to rDNA and over an extended region internal to telomeres.

### Telomere repeats alone are sufficient to promote CENP-A^Cnp1^ incorporation nearby

**Figure 4:**
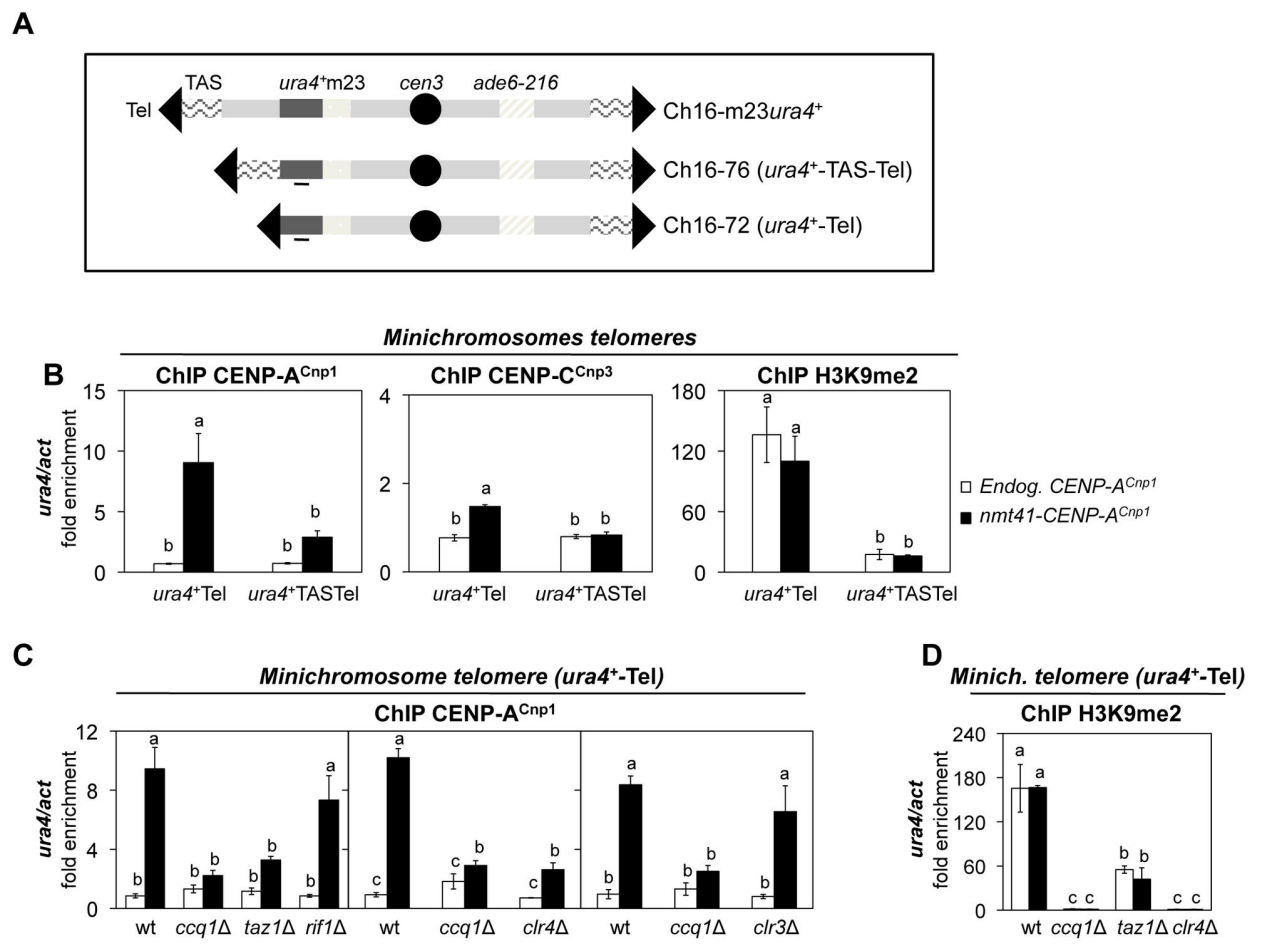
Terminal telomere repeats alone can attract CENP-A^Cnp1^ in cells expressing additional CENP-A^Cnp1^. (**A**) Schematic representation of Ch16-m23 minichromosome derivatives with the *ura4* ^+^ gene inserted 50 kb from the minichromosome end (Ch16-m23*ura4* ^+^) or adjacent to telomere-associated sequences at a telomere (*ura4* ^+^-TAS-Tel, Chs 16–76) or directly abutting terminal telomeric repeats (*ura4* ^+^-Tel, Chs 16–72). Black bar indicates the region at *ura4* ^+^ analyzed by ChIP-qPCR. (**B**) ChIP-qPCR of CENP-A^Cnp1^, CENP-C^Cnp3^ and H3K9me2 levels on *ura4* ^+^ in cells containing the *ura4* ^+^-Tel or *ura4* ^+^-TAS-Tel Ch16 minichromosomes and expressing endogenous (Endog. CENP-A^Cnp1^) or additional CENP-A^Cnp1^ levels (*nmt41*-CENP-A^Cnp1^) (**C**) ChIP-qPCR of CENP-A^Cnp1^ levels on *ura4* ^+^ in wild-type (wt) or mutant cells (*ccq1∆*, *taz1*∆, *clr4∆* and *clr3∆*) that contain the *ura4* ^+^-Tel or *ura4* ^+^-TAS-Tel Ch16 minichromosomes and express endogenous (Endog. CENP-A^Cnp1^) or additional (*nmt41*-CENP-A^Cnp1^) levels of CENP-A^Cnp1^ (**D**) ChIP-qPCR of CENP-A^Cnp1^ and H3K9me2 levels at *ura4* ^+^ in wild-type (wt) or mutant cells (*ccq1∆*, *taz1∆* and *clr4∆*) that contain the *ura4* ^+^-Tel and express endogenous (Endog. CENP-A^Cnp1^) or additional (*nmt41*-CENP-A^Cnp1^) levels of CENP-A^Cnp1^. For all ChIP analyses, enrichment on *ura4* ^+^ was normalized the signal obtained for the gene encoding actin (*act1* ^+^). Error bars indicate S.D. from 3 biological replicates. Mean values marked with different letter (a, b or c) indicate results significantly different from each other, as established by One Way ANOVA and Holm-Sidak test for multiple comparison (P<0.01).

The natural telomeres on fission yeast chromosomes 1 and 2 are composed of a terminal ~300 bp of terminal telomeric repeats (TTAC(A) GG(G_1-4_)) internal to which are copies of telomere associated sequence (TAS) repeat elements [21]. Part of the TAS regions share homology with centromeric *otr* repeats [22,23]. To determine if TAS or terminal repeats are required to attract CENP-A^Cnp1^ to domains of telomeric chromatin, we utilised versions of a well characterised minichromosome Ch16 in which new telomeres were created adjacent to a *ura4* ^+^ marker gene by telomere-mediated chromosome breakage at the *m23* locus [40]. On Chs 16–76, 1.2 kb of TAS DNA lies between the terminal telomeric repeat array and the *ura4* ^*+*^ gene (*ura4* ^+^ *-TAS-Tel*), whereas on Chs 16–72 the terminal telomere repeat array lies directly adjacent to the *ura4* ^*+*^ gene (*ura4* ^+^ *-Tel*) (Figure 4A). These minichromosomes are non-essential, allowing monitoring of events that might be lethal if they occurred on endogenous chromosomes. The placement of the *ura4* ^*+*^ gene next to telomeric sequences allows chromatin-mediated silencing to be monitored and provides unique primer sites for qPCR-ChIP analyses on the Ch16 minichromosome which is otherwise identical in sequence to a ~530 kb section of chromosome 3 encompassing *cen3*.

When additional CENP-A^Cnp1^ was expressed in cells bearing these minichromosomes, increased levels of CENP-A^Cnp1^ were detected on the *ura4* ^*+*^ gene. Significantly more CENP-A^Cnp1^ was detected on *ura4* ^*+*^ when it resided immediately adjacent to the terminal telomere repeat (*ura4* ^+^ *-Tel*) relative to when TAS sequences were included (*ura4* ^+^ *-TAS-Tel*) (Figure 4B and Figure S3A). Moreover, the CENP-C^Cnp3^ kinetochore protein was also enriched at *ura4* ^+^ *-Tel* in cells expressing additional CENP-A^Cnp1^ (Figure 4B and Figure S3A). The fact that CENP-A^Cnp1^ is detected on *ura4* ^+^ *-Tel* indicates that TAS elements are not required to attract CENP-A^Cnp1^ to telomeric regions. The diminished deposition of additional CENP-A^Cnp1^ on the *TAS*-adjacent *ura4* ^+^ could be due to an inhibitory effect of *TAS* or simply due to the difference in distance of *ura4* ^+^ from the telomeric repeats in Chs 16–72 versus Chs 16–76. Nonetheless, CENP-A^Cnp1^ and CENP-C^Cnp3^ are clearly attracted to telomeric adjacent regions independently of *TAS* elements.

Heterochromatin driven by H3K9 methylation coats the outer repeats at centromeres, a region containing the silent mating type genes, and the regions adjacent to telomeres. To determine whether the presence of heterochromatin influences CENP-A^Cnp1^ deposition at telomeres, anti-H3K9me2 ChIP was performed. Our analyses show that H3K9me2 is enriched on *ura4* ^+^ *-Tel*, but only weakly enriched on *ura4* ^+^ *-TAS-Tel* when compared to the enrichment found at outer repeats (Figure 4B and Figure S3B). Thus, CENP-A^Cnp1^ is incorporated in regions close to a telomere where high levels of H3K9me2 are also present.

The presence of an active *ura4* ^+^ gene (Ch16-m23-*ura4* ^+^) allows growth in the absence of uracil (–ura) and no growth on media containing counter-selective FOA (Figure S4). When placed next to telomeres the *ura4* ^+^ gene is transcriptionally silenced by telomeric heterochromatin as indicated by enhanced growth of Chs 16–72 (*ura4* ^+^ *-Tel*) and Chs 16–76 (*ura4* ^+^ *-TAS-Tel*) on FOA plates and/or reduced growth on –uracil plates (Figure S4). The degree of silencing is correlated with H3K9 methylation: *ura4* ^+^ *-TAS-Tel* exhibits low levels of H3K9me2 and weak silencing whereas *ura4* ^+^ *-Tel* displays high levels of H3K9me2 and stronger silencing (Figure 4B and Figure S4). The lack of heterochromatin may affect the ability of *ura4* ^+^ *-TAS-Tel* to incorporate CENP-A^Cnp1^. Indeed expression of additional CENP-A^Cnp1^ enhanced silencing of telomere-adjacent *ura4* ^+^ *-Tel* (reduced growth on –ura plates: Figure S4) which is consistent with the increase in CENP-A^Cnp1^ levels detected (Figure 4B), but did not enhance silencing of TAS-adjacent *ura4* ^+^ *-TAS-Tel*.

### The incorporation of CENP-A^Cnp1^ near telomeres requires Ccq1 and Clr4

Telomeres are bound by several proteins that control telomere length homeostasis, by regulating the recruitment of telomerase [24]. Loss of Taz1, Rap1, or Rif1 results in elongated telomeres [25,27,28] whereas loss of Ccq1, which is involved in telomerase recruitment, results in shortened telomeres [29,34,35]. Ccq1 also associates with the SHREC complex that contains the Clr3 HDAC, and cooperates with Taz1 in recruiting SHREC to telomeres [30].

To investigate the requirements for the incorporation of CENP-A^Cnp1^ near telomeres additional CENP-A^Cnp1^ was expressed in mutants that affect telomere structure and function. The level of CENP-A^Cnp1^ associated with *ura4* ^+^ *-Tel* on the minichromosome was markedly reduced in cells lacking Clr4 or Ccq1 relative to wild-type cells (Figure 4C). Cells lacking Rif1 or Clr3 incorporated CENP-A^Cnp1^ on *ura4* ^+^ *-Tel* at levels similar to wild-type (Figure 4C), whereas *taz1Δ* cells displayed intermediate levels of CENP-A^Cnp1^ incorporation. In all cell types analysed (wt, *ccq1∆*, *rif1∆*, *taz1∆*, *clr4∆*, *clr3∆*) the observed incorporation of CENP-A^Cnp1^ on the centromeric outer repeats and the central core was unaffected (Figures S5A and S5B), thus indicating that Clr4 and Ccq1, and to a lesser extent Taz1, play a specific role in promoting CENP-A^Cnp1^ deposition near telomeres.

ChIP was also performed to determine the level of H3K9me2 at *ura4* ^+^ *-Tel* in these different backgrounds. As expected, all H3K9me2 was lost in *clr4∆* cells (Figure 4D). In *taz1∆* cells there was some reduction in the level of H3K9me2 at *ura4* ^+^ *-Tel*, which might be a consequence of having highly elongated telomeres. Surprisingly, in *ccq1∆* cells, H3K9me2 was completely lost from *ura4* ^+^ *-Tel* but retained on centromere repeats (Figures 4D and S5C). This observation suggests that the incorporation of CENP-A^Cnp1^ at telomeres is linked to the loss of telomeric H3K9 methylation.

### A chromosome end is not required to attract additional CENP-A^Cnp1^

**Figure 5:**
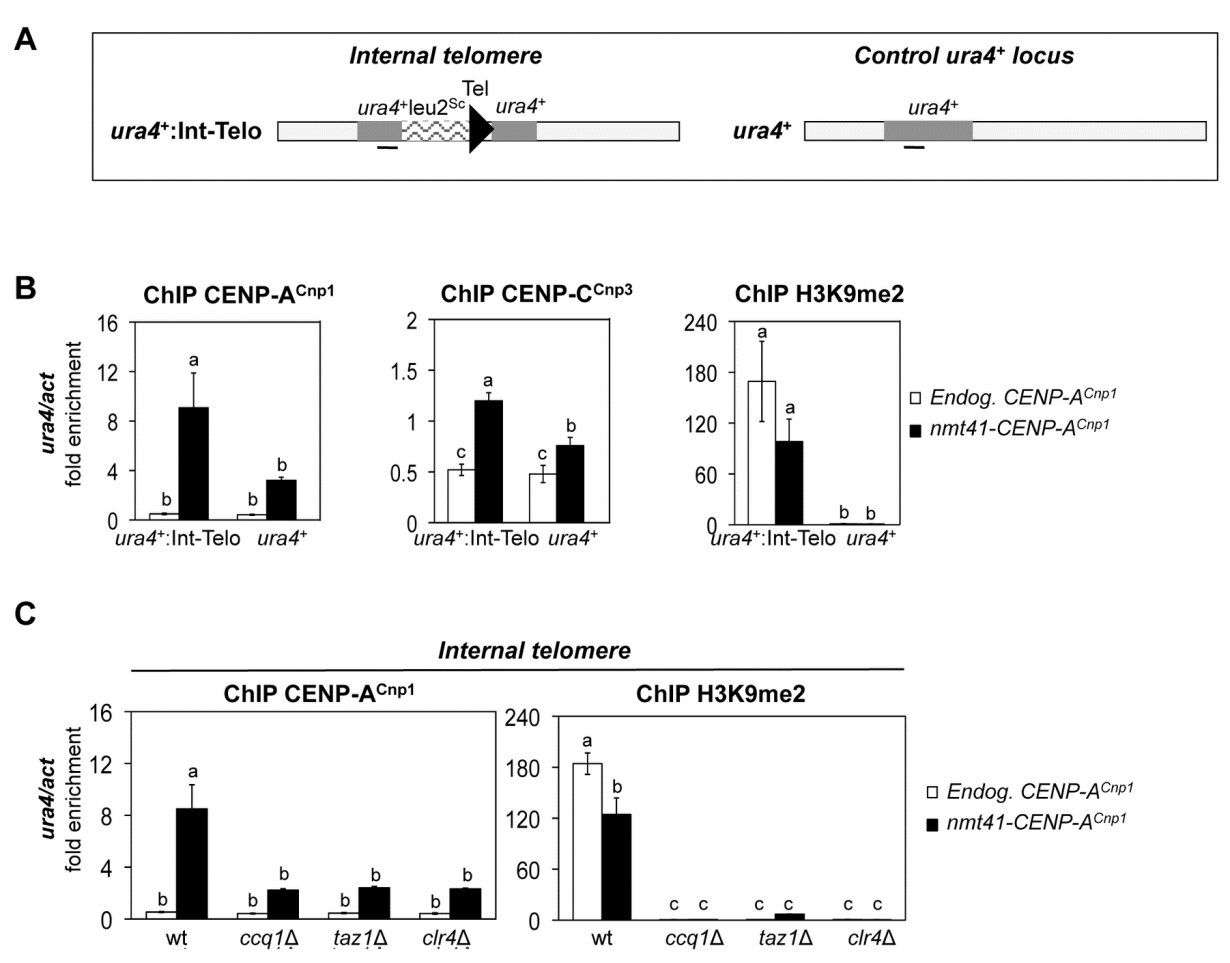
Telomeric repeat arrays promote CENP-A^Cnp1^ deposition when inserted at an internal chromosomal location. (**A**) Schematic representation of the genomic region of *S. pombe* chromosome III where an array of telomeric repeats was inserted into the *ura4* ^+^ gene (Internal telomere: *ura4* ^+^: Int-Telo) [41]. Black bar indicates the region at *ura4* ^+^ analyzed by ChIP-qPCR. (**B**) ChIP-qPCR of CENP-A^Cnp1^, CENP-C^Cnp3^ and H3K9me2 levels on *ura4* ^+^ in control cells (*ura4* ^+^) or cells with telomeric repeats integrated at *ura4* ^+^ (*ura4* ^*+*^: Int-Telo) and that express endogenous (Endog. CENP-A^Cnp1^) or additional (*nmt41*-CENP-A^Cnp1^) levels of CENP-A^Cnp1^. (**C**) ChIP analysis of CENP-A^Cnp1^ and H3K9me2 levels on *ura4* ^*+*^: Int-Telo (qPCR1) in wild-type (wt) or mutant cells (*ccq1∆*, *taz1*∆, and *clr4∆*) expressing endogenous (Endog. CENP-A^Cnp1^) or additional (*nmt41*-CENP-A^Cnp1^) levels of CENP-A^Cnp1^. For all ChIP analyses, enrichment on *ura4* ^+^ was normalized to the signal obtained for the gene encoding actin (*act1* ^+^). Error bars indicate S.D. from 3 biological replicates. Mean values marked with different letter (a, b or c) indicate results significantly different from each other, as established by One Way ANOVA and Holm-Sidak test for multiple comparison (P<0.01).

Chromosome ends have a distinct structure that recruits a plethora of factors that could influence the incorporation of CENP-A^Cnp1^ adjacent to telomere repeats. To determine whether the deposition of CENP-A^Cnp1^ near telomeric repeats requires proximity to a chromosome end we examined the association of CENP-A^Cnp1^ adjacent to a 300 bp array of telomeric repeats inserted adjacent to the *ura4* ^*+*^ gene within a chromosome arm (Figure 5A [41]). In cells expressing additional CENP-A^Cnp1^, elevated levels of CENP-A^Cnp1^ and CENP-C^Cnp3^ were readily detected adjacent to these interstitial telomere repeats but not the control *ura4* ^+^ locus alone (Figure 5B), whereas the levels of CENP-A^Cnp1^ on the centromeric outer repeats and the central core was similar in both strains (Figure S6). Moreover, the incorporation of CENP-A^Cnp1^ again correlated with the presence of H3K9me2 in that H3K9me2 was readily detected at *ura4* ^+^: Int-Telo (Figure 5B). We conclude that proximity to a chromosome end is not required to promote CENP-A^Cnp1^ deposition.

We next examined the level of H3K9me2 and the incorporation of CENP-A^Cnp1^ near internal telomere repeats (*ura4* ^+^: Int-Telo) in mutant cells expressing additional CENP-A^Cnp1^. In *clr4∆*, *taz1∆* and *ccq1∆* cells the level of CENP-A^Cnp1^ at *ura4* ^+^: Int-Telo was dramatically reduced compared to the wild type control and the reduced deposition of CENP-A^Cnp1^ correlates with the loss of H3K9me2 at *ura4* ^+^: Int-Telo observed in these mutants (Figure 5C). CENP-A^Cnp1^ and H3K9me2 levels at centromeres are not affected in mutants with defective telomere proteins (Figure S7). These analyses suggest that the telomere binding proteins promote the formation of H3K9me-dependent heterochromatin near telomeric repeats and, as at centromeres, this heterochromatin permits the incorporation of surplus CENP-A^Cnp1^.

### Additional CENP-A^Cnp1^ is attracted independently of heterochromatin to regions capable of neocentromere formation

**Figure 6:**
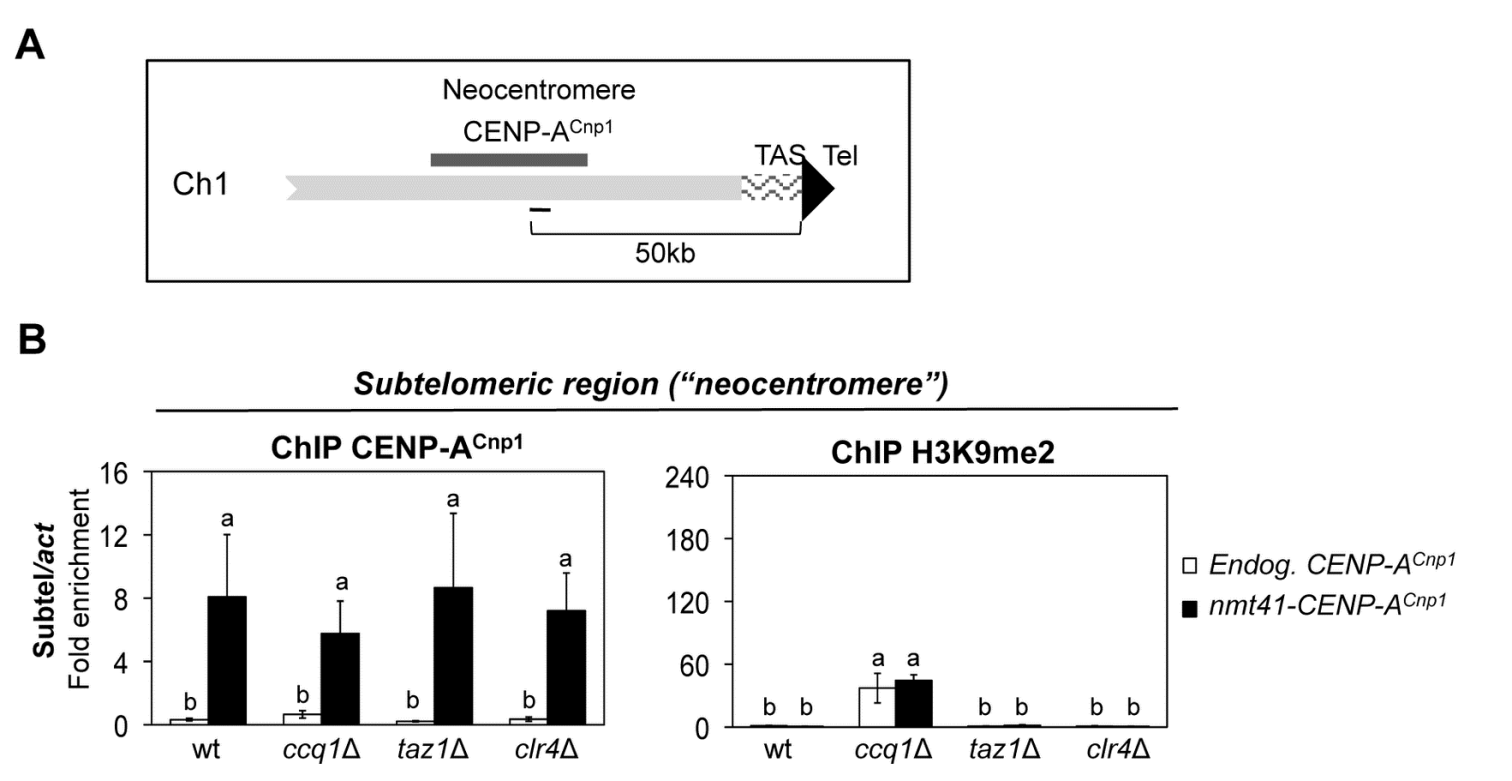
CENP-A^Cnp1^ is deposited on subtelomeric regions of natural chromosomes independently of heterochromatin. (**A**) Schematic representation of the right subtelomeric region of *S. pombe* chromosome I that has been shown to form neocentromeres (thick black line) [6]. Thin black line marks the region analyzed by ChIP-qPCR. (**B**) ChIP-qPCR of CENP-A^Cnp1^ and H3K9me2 levels associated with the subtelomeric region of chromosome I in wild-type (wt) or mutant cells (*ccq1∆*, *taz1*∆, and *clr4∆*) expressing endogenous (Endog. CENP-A^Cnp1^) or additional (*nmt41*-CENP-A^Cnp1^) levels of CENP-A^Cnp1^. Enrichment at the sub-telomere region (Subtel) was normalized to the signal obtained for the gene encoding actin (*act1* ^+^). Error bars indicate S.D. from 3 biological replicates. Mean values marked with different letter (a, b or c) indicate results significantly different from each other, as established by One Way ANOVA and Holm-Sidak test for multiple comparison (P<0.05).

Fission yeast neocentromeres have been shown to form over a ~ 40 kb domain that is positioned 40-60 kb from chromosome ends [6]. These neocentromeres overlap with the 100 kb region near telomeres where we observe incorporation of CENP-A^Cnp1^ when expressed at elevated levels (Figure 6A and Figure S2A). In contrast to the situation at synthetic telomeres at the ends of minichromosomes or internal telomere repeat arrays, CENP-A^Cnp1^ remains detectable in these neocentromere regions in all mutants analysed, including *clr4∆* cells where H3K9 methylation and heterochromatin is completely lost (Figure 6B [23]). Thus, the incorporation of CENP-A^Cnp1^ over these potential neocentromere regions is not dependent on the presence of heterochromatin. It is likely that endogenous sub-telomeric regions possess features that attract CENP-A^Cnp1^ by a route that is independent of heterochromatin and telomere-binding proteins, although these features are also able to attract CENP-A^Cnp1^.

## Discussion

In this study we set out to investigate what factors influence the sites where CENP-A can be incorporated when its levels are elevated. Normally CENP-A is deposited exclusively at centromeres where it forms the basis for assembly of the kinetochore. However, there is strong evidence that CENP-A incorporation can be epigenetically determined and that the normally preferred centromeric sequences are neither necessary nor sufficient for CENP-A deposition in certain circumstances. By expressing additional CENP-A^Cnp1^ in fission yeast we aimed to gain insight into which genomic regions, apart from centromeres, are preferred. This could provide clues to the enigma of neocentromere formation. What constitutes pre-centromeric sites i.e. what features are associated with the potential locations of neocentromeres? Is sequence important or chromatin context or both?

Unlike in *S. cerevisiae* and *Drosophila* , CENP-A^Cnp1^ can be overexpressed in fission yeast and thus its levels do not appear to be subject to tight regulation by ubiquitin-mediated degradation [14,15,42]. When the levels of CENP-A^Cnp1^ are raised by a modest amount we find that CENP-A^Cnp1^ accumulates at subtelomeric and pericentromeric regions. Thus, as has been demonstrated in some other species, there is flexibility with respect to the chromosomal locations at which fission yeast CENP-A^Cnp1^ assembles. The CENP-C^Cnp3^ kinetochore protein also becomes localised at some sites where additional CENP-A^Cnp1^ is incorporated, suggesting that these sites have the potential to become functional ectopic kinetochores. The toxicity associated with high levels of CENP-A^Cnp1^ overexpression (Figure 1A) might result from formation of ectopic kinetochores and/or global effects on transcriptional regulation [16]. In *Drosophila* , overexpression of CENP-A^CID^ also results in recruitment of CENP-C and formation of ectopic kinetochores near telomeres and pericentromeric regions [8,9]. As in *S. pombe*, heterochromatin influences recruitment of CENP-A^CID^ in *Drosophila* . In a recent study in *Saccharomyces cerevisiae*, overexpression of CENP-A^Cse4^ led to its incorporation at additional regions of the genome, termed Centromere-Like-Regions (CLRs). Although budding yeast lacks methyl-H3K9 heterochromatin, many CLRs were found in the regions surrounding centromeres [43]. CENP-A proteins also show a preference for transcribed regions in *S. cerevisiae* and *S. pombe* [16,17,43,44]

To investigate the preferential incorporation of additional CENP-A^Cnp1^ at subtelomeric regions we utilised minichromosomes with synthetic telomeres. In cells with elevated CENP-A^Cnp1^, CENP-A^Cnp1^ is incorporated on *ura4* ^*+*^ placed adjacent to a terminal array of telomeric repeats (*ura4* ^+^ *-Tel*) that lack an intervening TAS element. Thus, TAS sequences are not required to attract CENP-A^Cnp1^ to telomeric regions. As these Ch16-based minichromosomes also lack the 100 kb sub-telomeric region where neocentromeres form, we conclude that these regions are not necessary to attract CENP-A^Cnp1^ to telomeres (but see below). Our finding that telomere repeats inserted at an internal chromosomal location can also attract CENP-A^Cnp1^ indicates that proximity to a chromosome end is not a requirement; thus the specialised structure that caps chromosome ends and involves many telomere associated proteins is not needed. At natural telomeres, heterochromatin formation is dependent on Clr4 methyltransferase which is recruited directly by terminal telomere repeats, and by the action of RNAi on regions of the subtelomeric repeats that are homologous to centromeric outer repeats [23]. At the synthetic *ura4* ^+^ *-Tel* telomere and *ura4* ^+^: Int-Telo internal telomere repeats, the heterochromatin-specific mark H3K9me2 is dependent on both Clr4 and Ccq1. However, the other components of the SHREC complex do not seem to be involved as lack of the HDAC subunit Clr3 does not affect CENP-A^Cnp1^ deposition (Figure 4C). Thus, unexpectedly, we find that Ccq1 is required to induce H3K9me2 at arrays of TTAC(A) GG(G_1-4_) telomere repeats regardless of their location or the presence of TAS elements. These analyses suggest that Ccq1 has a role in recruiting Clr4 via telomeric repeats and that it is the resulting heterochromatin that promotes the incorporation of CENP-A^Cnp1^.

Consistent with a role for heterochromatin in promoting CENP-A^Cnp1^ deposition we also detected CENP-A^Cnp1^ accumulation on the heterochromatic outer repeats at centromeres in cells expressing additional CENP-A^Cnp1^. However, unlike at telomeres, when heterochromatin is lost (*clr4∆*), CENP-A^Cnp1^ is still incorporated on these outer repeats. It is likely that at centromeres the presence of CENP-A^Cnp1^ chromatin, and associated loading factors promotes the incorporation of this additional CENP-A^Cnp1^. Barrier function has been ascribed to tRNA genes that lie between heterochromatin and CENP-A^Cnp1^ chromatin domains at centromeres [38]. The presence of CENP-A^Cnp1^ on the outer repeats in cells expressing elevated levels suggests that these barriers can be overcome so that CENP-A^Cnp1^ can spread beyond the tRNA genes into the flanking heterochromatin region (Figure 2A Figures S2A and S2B).

It is clear that heterochromatin and telomere-associated proteins are required to recruit additional CENP-A^Cnp1^ at synthetic telomeres. However, the situation is more complex at natural telomeric regions. Our ChIP analyses indicate that additional CENP-A^Cnp1^ is incorporated in a 100 kb sub-telomeric domain in wild-type cells. These regions overlap with the regions that have been shown to become neocentromeres [6]. Although neocentromere formation is reduced in heterochromatin-deficient mutants, it is not abolished. Our analyses indicate that heterochromatin is not required for incorporation of CENP-A^Cnp1^ within this sub-telomeric domain (Figure 6). Thus, this region of the genome appears to have an inherent ability to attract CENP-A^Cnp1^. Particular combinations of features, such as proximity to a chromosome end, telomeric repeat sequences, telomere-specific proteins, heterochromatin, subtelomeric regions with homology to the centromeric outer repeats, telomere associated sequences (TAS), association with the nuclear periphery, or even sequence-specific properties of these 100 kb sub-telomeric regions may be responsible for attracting CENP-A^Cnp1^ to regions near chromosome ends.

In *S. pombe*, heterochromatin is required for establishment, but not maintenance, of CENP-A^Cnp1^ chromatin on adjacent central domain sequences [18]. Here, we have investigated the involvement of heterochromatin in attracting CENP-A^Cnp1^ to other regions of the genome and find that it is sufficient but not necessary for ectopic localisation of CENP-A^Cnp1^. In *Drosophila* , overproduced CENP-A^CID^ is also attracted to heterochromatic loci [9]. How does heterochromatin promote deposition of CENP-A^Cnp1^ on adjacent/overlapping sequences? One possibility is that the chromatin environment that heterochromatin engenders – such as histone hypoacetylation or transcriptional features - influences CENP-A^Cnp1^ deposition. Alternatively, CENP-A^Cnp1^ incorporation might be a consequence of the compartmentalisation of heterochromatin. Centromeres are clustered at the spindle pole body (SPB), whilst telomeres are located at the nuclear periphery. This tethering constrains the nuclear volume that they explore, increasing the likelihood that they will come into contact with centromeres. It is likely that a domain around the centromeres / SPB contains high concentrations of CENP-A^Cnp1^ and assembly factors. Thus, telomeres and other heterochromatic loci may be favoured sites for CENP-A^Cnp1^ assembly simply because they frequently come into contact with centromeres. Both centromeres and subtelomeres interact with the same inner membrane protein, Man1, at the nuclear periphery [45]. These interactions could favour CENP-A deposition at subtelomeres.

With the exception of organisms with holocentric chromosomes, it is essential that only one kinetochore is assembled per chromosome; dicentric chromosomes are inherently unstable. On the other hand, the existence of neocentromeres indicates that CENP-A is able to assemble on non-centromeric sequences. Indeed, the formation of neocentromeres is thought to be important in genome evolution and speciation [46]. Thus CENP-A must have the ability to be normally specific in its deposition requirements, but occasionally promiscuous. The factors that influence the constraints and permissiveness of CENP-A deposition are clearly complex, but our study indicates that heterochromatin is able to promote CENP-A deposition.

## Materials and Methods

### Cell growth and manipulation

Standard genetic and molecular techniques were followed. Fission yeast methods were as described [47]. For the strains used in the experiments, see Supporting information (Table S1). The CENP-A^Cnp1^ open reading frame was cloned into three plasmids containing different strengths of the thiamine repressible *nmt* promoter, pREP81X (pREP81-cnp1+), pREP41X (pREP41-cnp1+ [16], or pREP3X (pREP3-cnp1+). The constructs were linearized using *Mlu*I and integrated at *ars1* locus. The Ch16 minichromosomes derivatives used in this work were described in [40]. The strain carrying an array of telomeric repeats inserted adjacent to the *ura4* ^+^ gene within chromosome arm (*ura4* ^+^: Int-telo) is described elsewhere [41]

### Growth of cells overexpressing CENP-A^Cnp1^ for ChIP analyses

Cells expressing additional CENP-A^Cnp1^ from integrated pREP81-*cnp1* ^*+*^ (*nmt81-CENP-A* ^*Cnp1*^), pREP41-*cnp1* ^*+*^ (*nmt41-CENP-A* ^*Cnp1*^) or cells with integrated empty vector were initially grown in rich medium which contains thiamine to repress the expression of additional CENP-A^Cnp1^. Cells were grown in PMG liquid medium (with thiamine) at 25^°^C and shifted to 36^°^C in PMG lacking thiamine for 24 h to allow CENP-A^Cnp1^ expression before ChIP analysis. Strains carrying Ch16 minichromosome derivatives were grown in media lacking adenine to select for the minichromosome.

### ChIP

ChIP was performed as described [36] using anti-CENP-A^Cnp1^ antibody (10 µl of anti-CENP-A^Cnp1^ antibody per 300 µl chromatin extract), anti-CENP-C^Cnp3^ antibody (10 µl of anti-CENP-C^Cnp3^ antibody per 300 µl chromatin extract) and anti-H3K9me2 antibody (1 µl of mAb 5.1.1 (a kind gift of Takeshi Urano antibody per 300 µl chromatin extract) and subsequently analyzed by quantitative PCR (qPCR) or by multiplex PCR. Table S2 lists primers used in qPCR and Table S3 lists primers used in multiplex PCR. Bars from ChIP-qPCR data represent the means +/- standard deviation (SD) from three independent biological replicates. Mean values marked with the same letter (a, b or c) indicate results not significantly different from each other, as established by one Way ANOVA and Holm-Sidak test for multiple comparisons (*P*, 0.01) using SigmaPlot 11 software (Systat Software Inc.)

### ChIP–chip

DNA was immunoprecipitated as described earlier using 10 µl of anti-CENP-A^Cnp1^ antibody per 100 µl chromatin extract [36]. For analysis on GeneChip *S. pombe* Tiling 1.0FR Arrays (Affymetrix) 5 mM dUTP was added to the second round of DNA amplification. Fragmentation, labelling and hybridization were performed by the Affymetrix core facility at Karolinska Institiutet (BEA) using standard protocols (http://www.affymetrix.com). Raw data from Affymetrix (.CEL format) were normalized with Affymetrix Tiling Analysis Software (TAS) v 1.1 and analyzed and visualized using Integrated Genome Browser (IGB [48],. Normalized signal of each probe (25nt) were obtained from Tiling Arrays Software (TAS; Affymetrix) according to [49]. For Figure 2, probe values were averaged to give one value for one genomic region.

The microarray data from this publication have been submitted to the GEO database [http://www.ncbi.nlm.nih.gov/geo/] and assigned the accession number GSE46427.

### Cytology

Immunolocalization was performed as described [50]. Cells were grown in PMG liquid medium (with thiamine) at 25^°^C and shifted to 36^°^C for 24 h to allow CENP-A^Cnp1^ expression before fixation. Cells were fixed for 7 min in 3.7% formaldehyde. The following antibodies were used: sheep CENP-A^Cnp1^ antiserum (1:2000) and Alexa-488-coupled donkey anti-sheep secondary antibody (1:1000) (Invitrogen – Life Technologies). Microscopy was performed as described [50] using a Zeiss Imaging 2 microscope (Zeiss, http://www.zeiss.com). Image acquisition was controlled using Metamorph software (Universal Imaging Corporation, http://www.moleculardevices.com). For comparison of cells expressing CENP-A^Cnp1^ from different promoters, identical exposures are used for imaging of all cells stained for CENP-A^Cnp1^ in any given set. Images are not autoscaled, instead they are scaled relative to the brightest images in the set in order to reflect relative signal intensity obtained from the different strengths of promoter. Thus, images are scaled relative to the *nmt81*-CENP-A^Cnp1^ cells in Figure 1, and relative to *nmt1*-CENP-A^Cnp1^ cells in Figure S1.

## Supporting Information

Figure S1 CENP-A^Cnp1^ overexpression in *S. pombe* cells. (**A**) CENP-A^Cnp1^ can be detected when overexpressed from the *nmt1* promoter. Adapted from Castillo et al., 2007 (Figure S3). Western analyses of extracts from wild-type cells overexpressing CENP-A^Cnp1^ from *nmt81* (81xC), *nmt41* (41xC) or *nmt1* (3xC) promoter to give low, medium and high expression levels compared with cells containing endogenous levels (3x). (**B**) Western analysis of GFP-CENP-A^Cnp1^ levels in wt and *spt16-18* cells. From Choi et al., 2012 (Figure 1D). Western analysis of GFP-CENP-A^Cnp1^ levels in wt and *spt16-18* cells expressing GFP-CENP-A^Cnp1^ under endogenous, *nmt81* or *nmt41* promoter (upper panel). The intensities of GFP-CENP-A^Cnp1^ and TAT-1 (alpha-tubulin) signals were measured using LICOR Odyssey Infrared Imaging System software (Li-COR Bioscience) and the relative intensities of GFP-CENP-A^Cnp1^/TAT-1 were quantified (bottom panel). GFP-CENP-A^Cnp1^ was expressed for 24 h at 25°C before harvest. (**C**) Comparison of CENP-A^Cnp1^ localisation in cells expressing CENP-A^Cnp1^ from different strengths of *nmt* promoter. Immunolocalization of CENP-A^Cnp1^ in cells expressing endogenous levels of CENP-A^Cnp1^ or extra CENP-A^Cnp1^ expressed from the *nmt81, nmt41 or nmt1* promoters. Cells were grown in the absence of thiamine for 24 h at 36^o^C and stained with anti-CENP-A^Cnp1^ antibody (green) and DAPI (DNA: red). Images are displayed to indicate the relative signal intensity in cells expressing CENP-A^Cnp1^ from the four different promoters. Images have not been autoscaled. The images for endogenous (Endog.) and *nmt81* promoters therefore appear very faint compared to *nmt1*. Scale bar, 5 µm.
(PDF)

Figure S2 Additional CENP-A^Cnp1^ accumulates at subtelomeric and pericentromeric regions. (**A**) Genome browser view showing ChIP-chip occupancy profiles for wild-type (Endg.) levels in blue or additional CENP-A^Cnp1^ levels in orange (*nmt81-CENP-A^Cnp1^*) at centromeres, pericentromeric outer repeats (x2, x4, x9 indicate the number of repetitive elements) or subtelomeric / rDNA-adjacent regions. Data on the Y-axis are presented in linear scale and X-axis shows genome positions (distance from the chromosome end at telomeres and rDNA loci is indicated in kb). ChIP-PCR analysis of CENP-A^Cnp1^ association with pericentromeric outer repeats (**B**) or subtelomeric / rDNA-adjacent regions (**C**) expressing wild-type (Endog.) or additional (*nmt81*) levels of CENP-A^Cnp1^. Enrichment of pericentromeric outer repeats (A, *otr* primers), telomeric (B, primers reside 21.7 kb, 53 kb and 72 kb from the left telomere of chromosome I and 3.7 kb and 47.9 kb from the right end of chromosome II) and rDNA adjacent regions (primers reside 34.5 kb from the rDNA repeats on left arm of chromosome III) was assessed compared to actin (act) in immunoprecipitated (IP) relative to total (T) extract. (**D**) ChIP-qPCR analysis of CENP-A^Cnp1^ association with a subtelomeric region from the right telomere of chromosome I (53 kb from the end) expressing wild-type (Endog.) or additional (*nmt81-CENP-A^Cnp1^*) levels of CENP-A^Cnp1^. Enrichment on the sub-telomeric region was normalized to the signal obtained for the euchromatic gene encoding actin (*act1* ^+^). Error bars indicate S.D. from 3 biological replicates. Mean values marked with different letter (a or b) indicate results significantly different from each other, as established by One Way ANOVA and Holm-Sidak test for multiple comparison (P<0.01).
(PDF)

Figure S3 Enrichment of CENP-A^Cnp1^, CENP-C^Cnp3^ and H3K9me2 at centromeres in wild-type cells carrying Ch16 minichromosome derivatives. (**A**) ChIP-qPCR of CENP-A^Cnp1^ and CENP-C^Cnp3^ levels at *TM1* in cells containing the *ura4* ^+^-Tel or *ura4* ^+^-TAS-Tel Ch16 minichromosomes and expressing endogenous (Endog. CENP-A^Cnp1^) or additional (*nmt41*-CENP-A^Cnp1^) levels of CENP-A^Cnp1^. Enrichment is reported as the percentage of immunoprecipitated chromatin (% IP). Error bars indicate S.D. from 3 biological replicates. (**B**) ChIP-qPCR of H3K9me2 levels on centromeric otr *dgI* repeats in the same cells as in A. Enrichment on *dgI* was normalized to the signal obtained for the gene encoding actin (*act1* ^+^). Error bars indicate S.D. from 3 biological replicates. Mean values marked with the same letter (a) indicate results not significantly different from each other, as established by One Way ANOVA and Holm-Sidak test for multiple comparison (P<0.01).
(PDF)

Figure S4 Increased CENP-A^Cnp1^ levels enhance silencing of a u*ra4* ^+^ gene placed adjacent to telomeres. Serial dilution growth assay of wild-type cells expressing wild-type levels (Endog.) or moderate levels of additional CENP-A^Cnp1^ (*nmt41*-CENP-A^Cnp1^) grown at 36^°^C for three days. Cells containing the m23:*ura4* ^+^ (*ura4* ^+^ inserted 50 kb from the left telomere of Ch16), *ura4* ^+^-Tel or *ura4* ^+^-TAS-Tel Ch16 minichromosomes were plated on the indicated media: no leucine, adenine or no uracil, or with counter-selective 5-FOA added.
(PDF)

Figure S5 Enrichment of CENP-A^Cnp1,^ CENP-C^Cnp3^ and H3K9me2 at centromeres in wild-type and mutant cells carrying the Ch16 *ura4* ^+^-Tel minichromosome derivatives. ChIP-qPCR of CENP-A^Cnp1^ levels on centromeric otr *dgI* repeats (**A**) and at TM1 in the central region of centromere 1 (**B**) in wild-type (wt) and mutant cells (*ccq1*∆, *taz1*∆, *rif1*∆, *clr4*∆ and *clr3*∆) carrying the Chs 16–72 (*ura4*+-Tel) minichromosome and expressing endogenous (Endog. CENP-A^Cnp1^) or additional (*nmt41*-CENP-A^Cnp1^) CENP-A^Cnp1^ levels. (**C**) ChIP-qPCR of H3K9me2 levels on centromeric otr *dgI* repeats in wild-type (wt) and mutant cells (*ccq1*∆, *taz1*∆, *and clr4*∆) with the same configuration. Enrichment on *dgI* was normalized the signal obtained for the gene encoding actin (*act1* ^+^). Enrichment at *TM1* is reported as the percentage of immunoprecipitated chromatin (% IP). Error bars indicate S.D. from 3 biological replicates. Mean values marked with different letter (a, b or c) indicate results significantly different from each other, as established by One Way ANOVA and Holm-Sidak test for multiple comparison (P<0.01 in (A) and (B) or P<0.05 in (C).
(PDF)

Figure S6 Enrichment of CENP-A^Cnp1^ at centromeres in cells with telomere repeats inserted at the *ura4* ^+^ locus. ChIP-qPCR of CENP-A^Cnp1^ levels at TM1 in the central region of centromere 1 (**A**) and centromeric otr *dgI* repeats (**B**) in cells containing an array of telomeric repeats integrated at the u*ra4* ^+^ locus (*ura4* ^+^: Int-Telo) or control cells (*ura4* ^+^) and expressing endogenous (Endog. CENP-A^Cnp1^) or additional (*nmt41-CENP-A^Cnp1^*) levels of CENP-A^Cnp1^. Enrichment on *dgI* was normalized the signal obtained for the gene encoding actin (*act1* ^+^). Enrichment at *TM1* is reported as the percentage of immunoprecipitated chromatin (% IP). Error bars indicate S.D. from 3 biological replicates. Mean values marked with different letter (a or b) indicate results significantly different from each other, as established by One Way ANOVA and Holm-Sidak test for multiple comparison (P<0.01).
(PDF)

Figure S7 Enrichment of CENP-A^Cnp1^, and H3K9me2 at centromeres in wild-type cells and mutants that affect telomeric chromatin. (**A**) ChIP-qPCR of CENP-A^Cnp1^ levels at TM1 in the central region of centromere 1 and centromeric otr *dgI* repeats in wild-type (wt) and mutant cells (*ccq1*∆, *taz1*∆, and *clr4*∆) which contain the internal telomere (*ura4* ^*+*^: Int-Telo) and that express endogenous (Endog. CENP-A^Cnp1^) or additional (*nmt41-CENP-A^Cnp1^*) levels of CENP-A^Cnp1^. (**B**) ChIP-qPCR of H3K9me2 levels in the same cell backgrounds. Enrichment at *TM1* is reported as the percentage of immunoprecipitated chromatin (% IP). Enrichment on *dgI* was normalized the background signal obtained for the gene encoding actin (*act1* ^+^). Error bars indicate S.D. from 3 biological replicates. Mean values marked with different letter (a, b or c) indicate results significantly different from each other, as established by One Way ANOVA and Holm-Sidak test for multiple comparison (P<0.01).
(PDF)

Table S1 List of strains used in this study. (PDF)

Table S2 Primers used for qPCR. (PDF)

Table S3 Primers used for multiplex PCR. (PDF)
