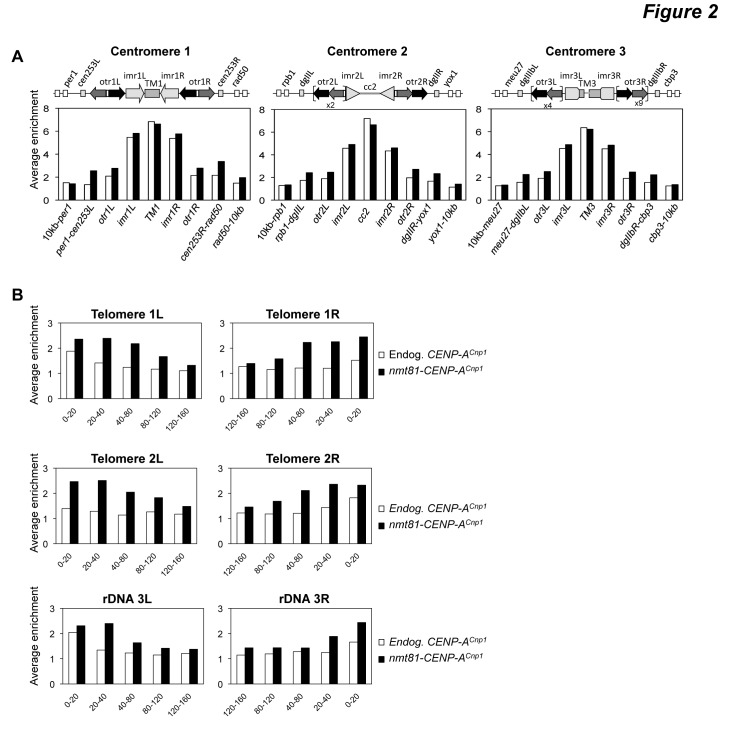# Correction: Telomeric Repeats Facilitate CENP-A^Cnp1^ Incorporation via Telomere Binding Proteins

**DOI:** 10.1371/annotation/c6430c38-0d7a-4b9f-aca9-7d8b212f9f09

**Published:** 2013-10-25

**Authors:** Araceli G. Castillo, Alison L. Pidoux, Sandra Catania, Mickaël Durand-Dubief, Eun Shik Choi, Georgina Hamilton, Karl Ekwall, Robin C. Allshire

Part of Figure 2 was cropped out during the production process. Please see the corrected Figure 2 here: 

**Figure:**