# Supplementary material for: Telomeric Repeats Facilitate CENP-ACnp1 Incorporation via Telomere Binding Proteins
Source: PLoS One. 2013 Jul 31;8(7):e69673. doi: 10.1371/journal.pone.0069673 (PMC3729655; doi:10.1371/journal.pone.0069673)
Supplement: Figure S1 — (A) CENP-ACnp1 can be detected when overexpressed from the nmt1 promoter. Adapted from Castillo et al., 2007 (Figure S3). Western analyses of extracts from wild-type cells overexpressing CENP-ACnp1 from nmt81 (81xC), nmt41 (41xC) or nmt1 (3xC) promoter to give low, medium and high expression levels compared with cells containing endogenous levels (3x). (B) Western analysis of GFP-CENP-ACnp1 levels in wt and spt16-18 cells. From Choi et al., 2012 (Figure 1D). Western analysis of GFP-CENP-ACnp1 levels in wt and spt16-18 cells expressing GFP-CENP-ACnp1 under endogenous, nmt81 or nmt41 promoter (upper panel). The intensities of GFP-CENP-ACnp1 and TAT-1 (alpha-tubulin) signals were measured using LICOR Odyssey Infrared Imaging System software (Li-COR Bioscience) and the relative intensities of GFP-CENP-ACnp1/TAT-1 were quantified (bottom panel). GFP-CENP-ACnp1 was expressed for 24 h at 25°C before harvest. (C) Comparison of CENP-ACnp1 localisation in cells expressing CENP-ACnp1 from different strengths of nmt promoter. Immunolocalization of CENP-ACnp1 in cells expressing endogenous levels of CENP-ACnp1 or extra CENP-ACnp1 expressed from the nmt81, nmt41 or nmt1 promoters. Cells were grown in the absence of thiamine for 24 h at 36oC and stained with anti-CENP-ACnp1 antibody (green) and DAPI (DNA: red). Images are displayed to indicate the relative signal intensity in cells expressing CENP-ACnp1 from the four different promoters. Images have not been autoscaled. The images for endogenous (Endog.) and nmt81 promoters therefore appear very faint compared to nmt1. Scale bar, 5 µm. (PDF) [file pone.0069673.s001.pdf]

Figure S1

A

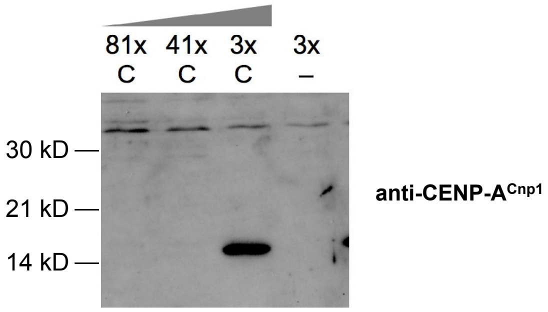

B

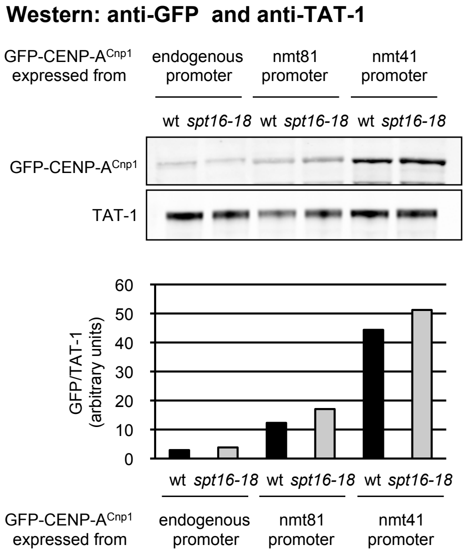

Figure S1. CENP-A^{Cnp1} overexpression in *S. pombe* cells.

(A) CENP-A^{Cnp1} can be detected when overexpressed from the *nmt1* promoter. Adapted from Castillo et al., 2007 (Figure S3). Western analyses of extracts from wildtype cells overexpressing CENP-A^{Cnp1} from *nmt81* (81xC), *nmt41* (41xC) or *nmt1* (3xC) promoter to give low, medium and high expression levels compared with cells containing endogenous levels (3x). **(B)** Western analysis of GFP-CENP-A^{Cnp1} levels in wt and *spt16-18* cells. From Choi et al., 2012 (Figure 1D). Western analysis of GFP-CENP-A^{Cnp1} levels in wt and *spt16-18* cells expressing GFP-CENP-A^{Cnp1} under endogenous, *nmt81* or *nmt41* promoter (upper panel). The intensities of GFP-CENP-A^{Cnp1} and TAT-1 (alpha-tubulin) signals were measured using LICOR Odyssey Infrared Imaging System software (Li-COR Bioscience) and the relative intensities of GFP-CENP-A^{Cnp1}/TAT-1 were quantified (bottom panel). GFP-CENP-A^{Cnp1} was expressed for 24 h at 25°C before harvest. **(C)** Comparison of CENP-A^{Cnp1} localisation in cells expressing CENP-A^{Cnp1} from different strengths of *nmt* promoter. Immunolocalization of CENP-A^{Cnp1} in cells expressing endogenous levels of CENP-A^{Cnp1} or extra CENP-A^{Cnp1} expressed from the *nmt81, nmt41 or nmt1* promoters. Cells were grown in the absence of thiamine for 24 h at 36°C and stained with anti-CENP-A^{Cnp1} antibody (green) and DAPI (DNA: red). Images are displayed to indicate the relative signal intensity in cells expressing CENP-A^{Cnp1} from the four different promoters. Images have not been autoscaled. The images for endogenous (Endog.) and *nmt81* promoters therefore appear very faint compared to *nmt1*. Scale bar, 5 mm.

Figure S1

C

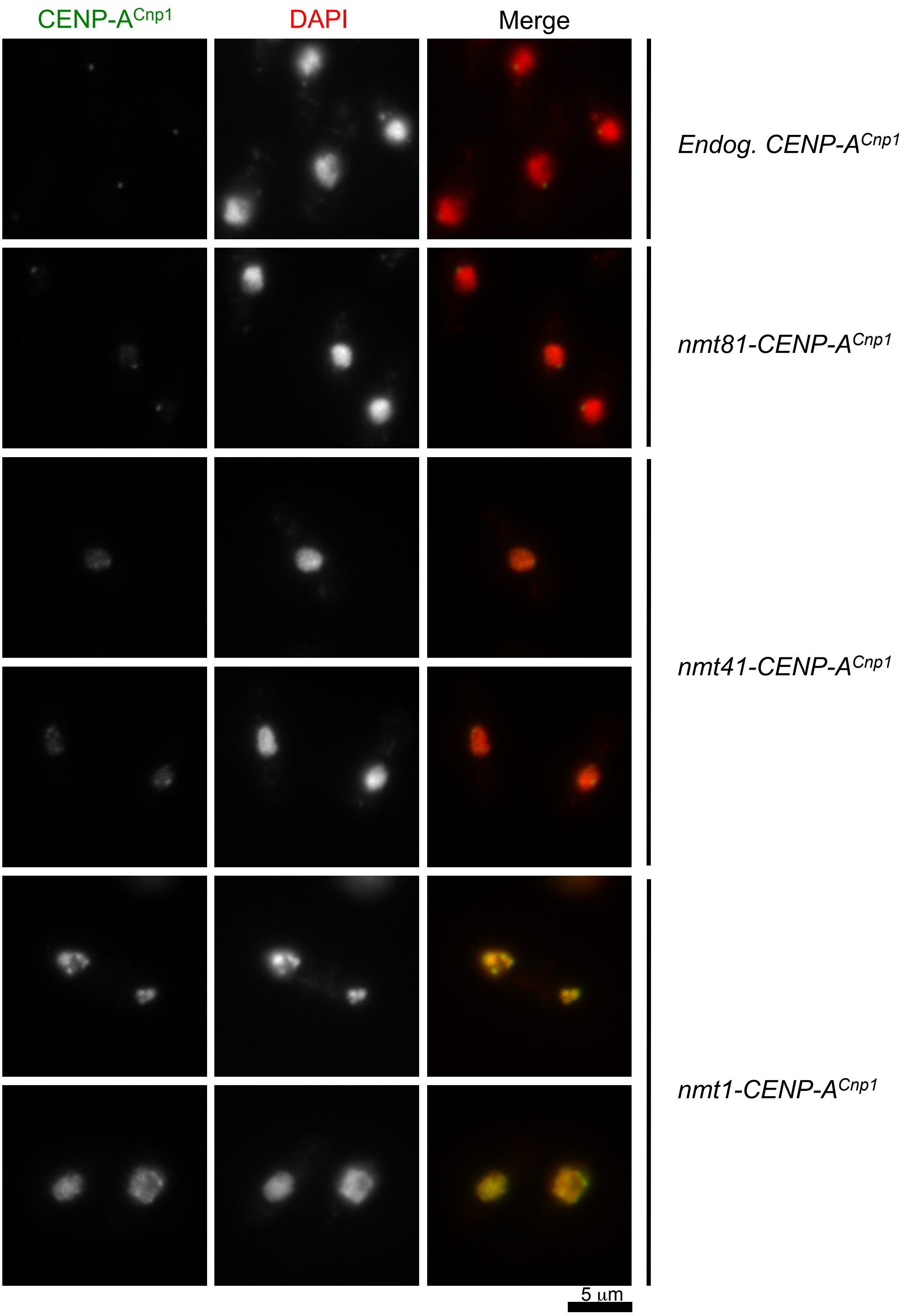

CENP-A^{Cnp1}
DAPI
Merge
Endog. CENP-A^{Cnp1}
nmt81-CENP-A^{Cnp1}
nmt41-CENP-A^{Cnp1}
nmt1-CENP-A^{Cnp1}
5 µm
