# Supplementary material for: Telomeric Repeats Facilitate CENP-ACnp1 Incorporation via Telomere Binding Proteins
Source: PLoS One. 2013 Jul 31;8(7):e69673. doi: 10.1371/journal.pone.0069673 (PMC3729655; doi:10.1371/journal.pone.0069673)
Supplement: Figure S2 — (A) Genome browser view showing ChIP-chip occupancy profiles for wild-type (Endg.) levels in blue or additional CENP-ACnp1 levels in orange (nmt81-CENP-ACnp1) at centromeres, pericentromeric outer repeats (x2, x4, x9 indicate the number of repetitive elements) or subtelomeric / rDNA-adjacent regions. Data on the Y-axis are presented in linear scale and X-axis shows genome positions (distance from the chromosome end at telomeres and rDNA loci is indicated in kb). ChIP-PCR analysis of CENP-ACnp1 association with pericentromeric outer repeats (B) or subtelomeric / rDNA-adjacent regions (C) expressing wild-type (Endog.) or additional (nmt81) levels of CENP-ACnp1. Enrichment of pericentromeric outer repeats (A, otr primers), telomeric (B, primers reside 21.7 kb, 53 kb and 72 kb from the left telomere of chromosome I and 3.7 kb and 47.9 kb from the right end of chromosome II) and rDNA adjacent regions (primers reside 34.5 kb from the rDNA repeats on left arm of chromosome III) was assessed compared to actin (act) in immunoprecipitated (IP) relative to total (T) extract. (D) ChIP-qPCR analysis of CENP-ACnp1 association with a subtelomeric region from the right telomere of chromosome I (53 kb from the end) expressing wild-type (Endog.) or additional (nmt81-CENP-ACnp1) levels of CENP-ACnp1. Enrichment on the sub-telomeric region was normalized to the signal obtained for the euchromatic gene encoding actin (act1 +). Error bars indicate S.D. from 3 biological replicates. Mean values marked with different letter (a or b) indicate results significantly different from each other, as established by One Way ANOVA and Holm-Sidak test for multiple comparison (P<0.01). (PDF) [file pone.0069673.s002.pdf]

Figure S2

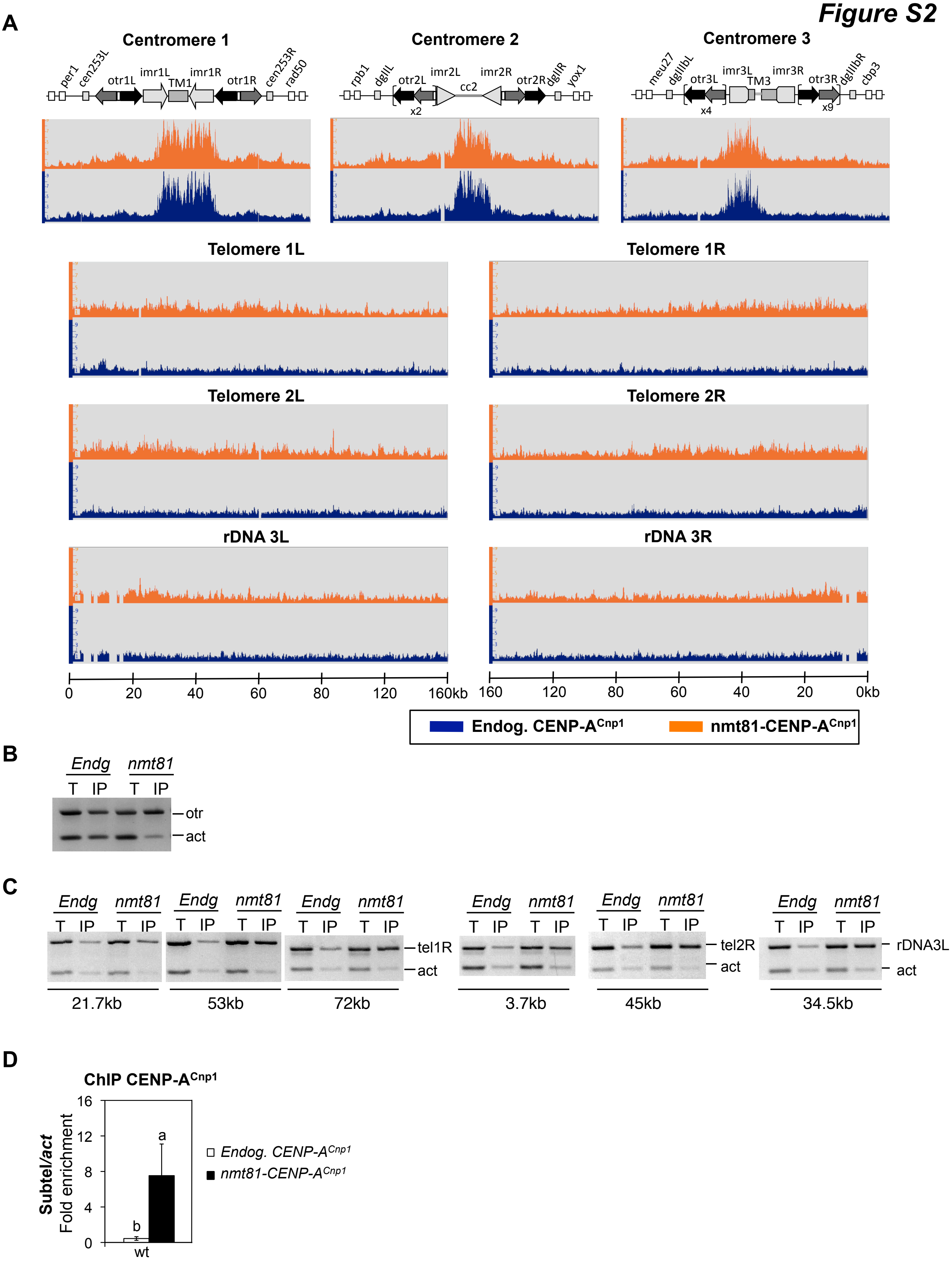
A
Centromere 1
per1
cen253L
otr1L
imr1L
TM1
imr1R
otr1R
cen253R
rad50
Centromere 2
rpb1
dgIIL
otr2L
imr2L
cc2
imr2R
otr2R
dgIIR
yox1
x2
Centromere 3
meu27
dgIIIbL
otr3L
imr3L
TM3
imr3R
otr3R
dgIIIbR
cbp3
x4
x9
Telomere 1L
Telomere 1R
Telomere 2L
Telomere 2R
rDNA 3L
rDNA 3R
0
20
40
60
80
120
160kb
160
120
80
60
40
20
0kb
Endog. CENP-A^{Cnp1}
nmt81-CENP-A^{Cnp1}
B
Endg
nmt81
T IP T IP
otr
act
C
Endg
nmt81
T IP T IP
tel1R
act
21.7kb
53kb
72kb
3.7kb
45kb
tel2R
rDNA3L
34.5kb
D
ChIP CENP-A^{Cnp1}
Subtel/act
Fold enrichment
16
12
8
4
0
a
b
wt
Endog. CENP-A^{Cnp1}
nmt81-CENP-A^{Cnp1}

Figure S2

Figure S2. Additional CENP-A^{Cnp1} accumulates at subtelomeric and pericentromeric regions.
(A) Genome browser view showing ChIP- chip occupancy profiles for wild-type (Endg.) levels in blue or additional CENP-A^{Cnp1} levels in orange (*nmt81-CENP-A^{Cnp1}*) at centromeres, pericentromeric outer repeats (x2, x4, x9 indicate the number of repetitive elements) or subtelomeric / rDNA-adjacent regions. Data on the Y-axis are presented in linear scale and X-axis shows genome positions (distance from the chromosome end at telomeres and rDNA loci is indicated in kb). ChIP-PCR analysis of CENP-A^{Cnp1} association with pericentromeric outer repeats (**B)** or subtelomeric / rDNA-adjacent regions **(C)** expressing wild-type (Endog.) or additional (*nmt81*) levels of CENP-A^{Cnp1}. Enrichment of pericentromeric outer repeats (A, *otr* primers), telomeric (B, primers reside 21.7 kb, 53 kb and 72 kb from the left telomere of chromosome I and 3.7 kb and 47.9 kb from the right end of chromosome II))and rDNA adjacent regions (primers reside 34.5 kb from the rDNA repeats on left arm of chromosome III) was assessed compared to actin (*act*) in immunoprecipitated (IP) relative to total (T) extract. **(D)** ChIP-qPCR analysis of CENP-A^{Cnp1} association with a subtelomeric region from the right telomere of chromosome I (53 kb from the end) expressing wild-type (Endog.) or additional (*nmt81-CENP-A^{Cnp1}*) levels of CENP-AC$^{np1.}$ Enrichment on the sub-telomeric region was normalized to the signal obtained for the euchromatic gene encoding actin (*act1+*). Error bars indicate S.D. from 3 biological replicates. Mean values marked with different letter (a or b) indicate results significantly different from each other, as established by One Way ANOVA and Holm-Sidak test for multiple comparison (P<0.01).
