# Supplementary material for: Telomeric Repeats Facilitate CENP-ACnp1 Incorporation via Telomere Binding Proteins
Source: PLoS One. 2013 Jul 31;8(7):e69673. doi: 10.1371/journal.pone.0069673 (PMC3729655; doi:10.1371/journal.pone.0069673)
Supplement: Figure S3 — (A) ChIP-qPCR of CENP-ACnp1 and CENP-CCnp3 levels at TM1 in cells containing the ura4 +-Tel or ura4 +-TAS-Tel Ch16 minichromosomes and expressing endogenous (Endog. CENP-ACnp1) or additional (nmt41-CENP-ACnp1) levels of CENP-ACnp1. Enrichment is reported as the percentage of immunoprecipitated chromatin (% IP). Error bars indicate S.D. from 3 biological replicates. (B) ChIP-qPCR of H3K9me2 levels on centromeric otr dgI repeats in the same cells as in A. Enrichment on dgI was normalized to the signal obtained for the gene encoding actin (act1 +). Error bars indicate S.D. from 3 biological replicates. Mean values marked with the same letter (a) indicate results not significantly different from each other, as established by One Way ANOVA and Holm-Sidak test for multiple comparison (P<0.01). (PDF) [file pone.0069673.s003.pdf]

# Figure S3

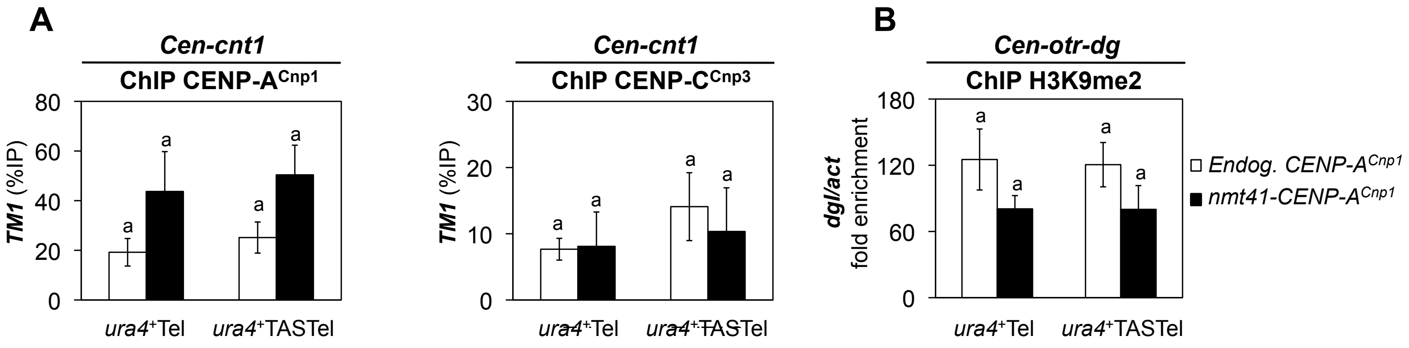


**Figure S3. Enrichment of CENP-A[Cnp1], CENP-C[Cnp3] and H3K9me2 at centromeres in wild-type cells carrying Ch16 minichromosome derivatives**

**(A)** ChIP-qPCR of CENP-A[Cnp1] and CENP-C[Cnp3] levels at *TM1* in cells containing the *ura4*+-Tel or *ura4*+-TAS-Tel Ch16 minichromosomes and expressing endogenous (Endog. CENP-ACnp1) or additional (*nmt41*-CENP-A[Cnp1]) levels of CENP-A[Cnp1]. Enrichment is reported as the percentage of immunoprecipitated chromatin (%IP). Error bars indicate S.D. from 3 biological replicates. **(B)** ChIP-qPCR of H3K9me2 levels on centromeric otr *dgI* repeats in the same cells as in **A**. Enrichment on *dgI* was normalized to the signal obtained for the gene encoding actin (*act1*+). Error bars indicate S.D. from 3 biological replicates. Mean values marked with the same letter (a) indicate results not significantly different from each other, as established by One Way ANOVA and Holm-Sidak test for multiple comparison (P<0.01).
