# Supplementary material for: Telomeric Repeats Facilitate CENP-ACnp1 Incorporation via Telomere Binding Proteins
Source: PLoS One. 2013 Jul 31;8(7):e69673. doi: 10.1371/journal.pone.0069673 (PMC3729655; doi:10.1371/journal.pone.0069673)
Supplement: Figure S4 — Serial dilution growth assay of wild-type cells expressing wild-type levels (Endog.) or moderate levels of additional CENP-ACnp1 (nmt41-CENP-ACnp1) grown at 36°C for three days. Cells containing the m23:ura4 + (ura4 + inserted 50 kb from the left telomere of Ch16), ura4 +-Tel or ura4 +-TAS-Tel Ch16 minichromosomes were plated on the indicated media: no leucine, adenine or no uracil, or with counter-selective 5-FOA added. (PDF) [file pone.0069673.s004.pdf]

Figure S4

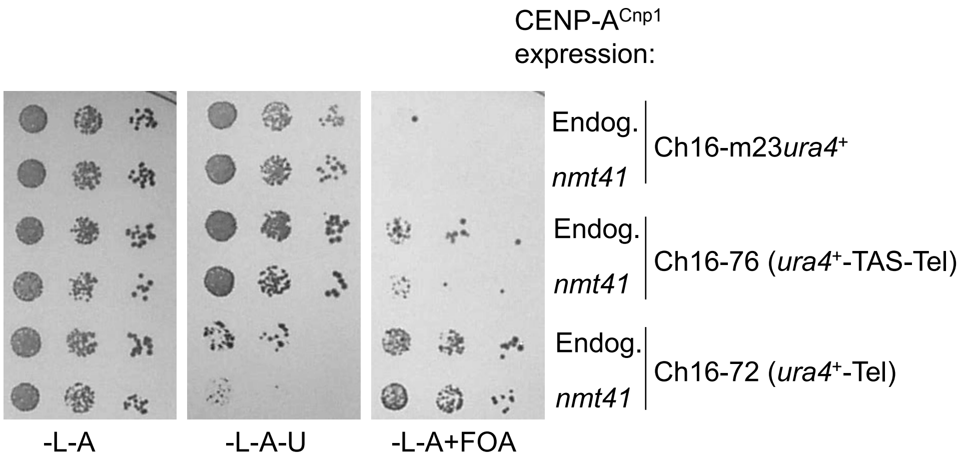

Figure S4. Increased CENP-A^{Cnp1} levels enhance silencing of a u*ra4*+ gene placed adjacent to telomeres.

Serial dilution growth assay of wild-type cells expressing wild-type levels (Endog.) or moderate levels of additional CENP-A^{Cnp1} (*nmt41*-CENP-A^{Cnp1}) grown at 36ºC for three days. Cells containing the m23:*ura4*+ (*ura4*+ inserted 50 kb from the left telomere of Ch16), *ura4*+-Tel or *ura4*+-TAS-Tel Ch16 minichromosomes were plated on the indicated media: no leucine, adenine or no uracil, or with counter-selective 5-FOA added.
