# Supplementary material for: Telomeric Repeats Facilitate CENP-ACnp1 Incorporation via Telomere Binding Proteins
Source: PLoS One. 2013 Jul 31;8(7):e69673. doi: 10.1371/journal.pone.0069673 (PMC3729655; doi:10.1371/journal.pone.0069673)
Supplement: Figure S5 — ChIP-qPCR of CENP-ACnp1 levels on centromeric otr dgI repeats (A) and at TM1 in the central region of centromere 1 (B) in wild-type (wt) and mutant cells (ccq1∆, taz1∆, rif1∆, clr4∆ and clr3∆) carrying the Chs 16–72 (ura4+-Tel) minichromosome and expressing endogenous (Endog. CENP-ACnp1) or additional (nmt41-CENP-ACnp1) CENP-ACnp1 levels. (C) ChIP-qPCR of H3K9me2 levels on centromeric otr dgI repeats in wild-type (wt) and mutant cells (ccq1∆, taz1∆, and clr4∆) with the same configuration. Enrichment on dgI was normalized the signal obtained for the gene encoding actin (act1 +). Enrichment at TM1 is reported as the percentage of immunoprecipitated chromatin (% IP). Error bars indicate S.D. from 3 biological replicates. Mean values marked with different letter (a, b or c) indicate results significantly different from each other, as established by One Way ANOVA and Holm-Sidak test for multiple comparison (P<0.01 in (A) and (B) or P<0.05 in (C). (PDF) [file pone.0069673.s005.pdf]

Figure S5

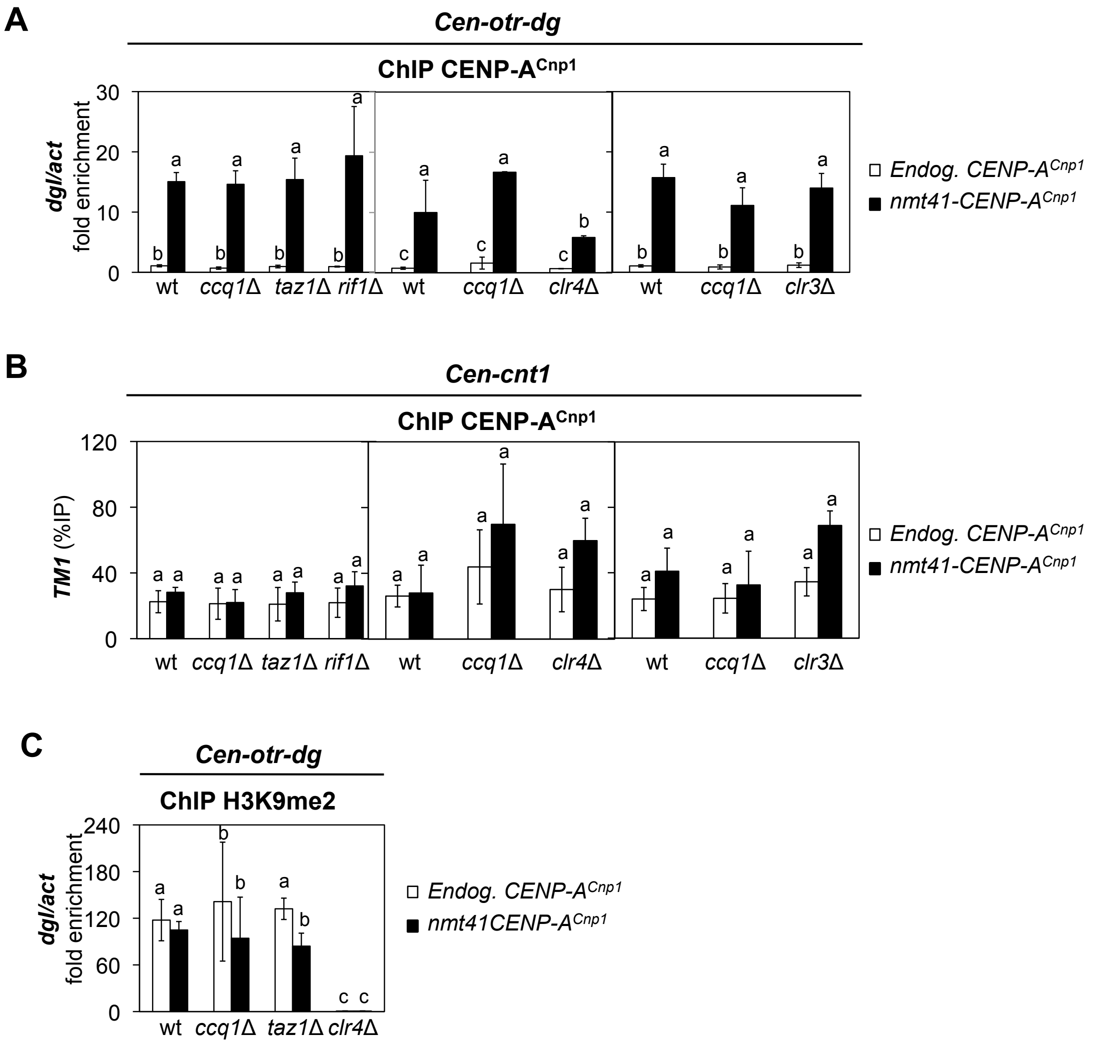

Figure S5. Enrichment of CENP-A^{Cnp1}, CENP-C^{Cnp3} and H3K9me2 at centromeres in wild-type and mutant cells carrying the Ch16 *ura4*+-Tel minichromosome derivatives.

ChIP-qPCR of CENP-A^{Cnp1} levels on centromeric otr *dgI* repeats **(A)** and at TM1 in the central region of centromere 1 **(B)** in wild-type (wt) and mutant cells (*ccq1*Δ, *taz1*Δ, *rif1*Δ, *clr4*Δ and *clr3*Δ) carrying the Ch16-72 (*ura4*+-Tel) minichromosome and expressing endogenous (Endog. CENP-A^{Cnp1}) or additional (*nmt41*-CENP-A^{Cnp1}) CENP-A^{Cnp1} levels. **(C)** ChIP-qPCR of H3K9me2 levels on centromeric otr *dgI* repeats in wild-type (wt) and mutant cells (*ccq1*Δ, *taz1*Δ, *and clr4*Δ) with the same configuration. Enrichment on *dgI* was normalized the signal obtained for the gene encoding actin (*act1*+). Enrichment at *TM1* is reported as the percentage of immunoprecipitated chromatin (%IP). Error bars indicate S.D. from 3 biological replicates. Mean values marked with different letter (a, b or c) indicate results significantly different from each other, as established by One Way ANOVA and Holm-Sidak test for multiple comparison ($P<0.01$ in (A) and (B) or $P<0.05$ in (C).
