# Supplementary material for: Telomeric Repeats Facilitate CENP-ACnp1 Incorporation via Telomere Binding Proteins
Source: PLoS One. 2013 Jul 31;8(7):e69673. doi: 10.1371/journal.pone.0069673 (PMC3729655; doi:10.1371/journal.pone.0069673)
Supplement: Figure S6 — ChIP-qPCR of CENP-ACnp1 levels at TM1 in the central region of centromere 1 (A) and centromeric otr dgI repeats (B) in cells containing an array of telomeric repeats integrated at the ura4 + locus (ura4 +: Int-Telo) or control cells (ura4 +) and expressing endogenous (Endog. CENP-ACnp1) or additional (nmt41-CENP-ACnp1) levels of CENP-ACnp1. Enrichment on dgI was normalized the signal obtained for the gene encoding actin (act1 +). Enrichment at TM1 is reported as the percentage of immunoprecipitated chromatin (% IP). Error bars indicate S.D. from 3 biological replicates. Mean values marked with different letter (a or b) indicate results significantly different from each other, as established by One Way ANOVA and Holm-Sidak test for multiple comparison (P<0.01). (PDF) [file pone.0069673.s006.pdf]

Figure S6

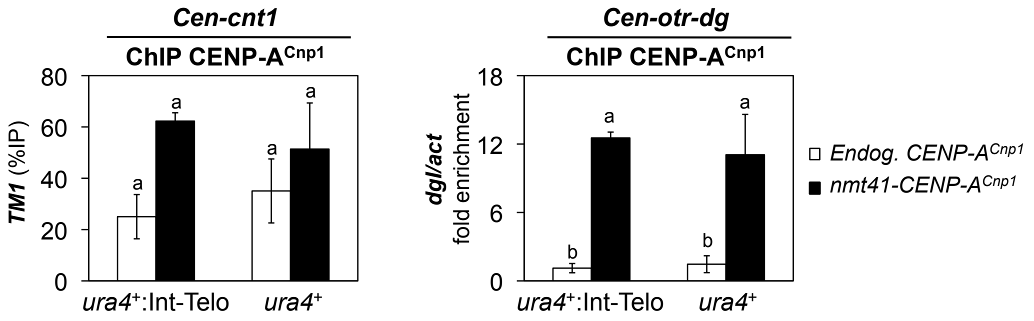

Figure S6. Enrichment of CENP-A^{Cnp1} at centromeres in cells with telomere repeats inserted at the *ura4*+ locus

ChIP-qPCR of CENP-A^{Cnp1} levels at TM1 in the central region of centromere 1 **(A)** and centromeric otr *dgI* repeats **(B)** in cells containing an array of telomeric repeats integrated at the u*ra4*+ locus (*ura4*+:Int-Telo) or control cells (*ura4*+) and expressing endogenous (Endog. CENP-A^{Cnp1}) or additional (*nmt41-CENP-A^{Cnp1}*) levels of CENP-A^{Cnp1}. Enrichment on *dgI* was normalized the signal obtained for the gene encoding actin (*act1*+). Enrichment at *TM1* is reported as the percentage of immunoprecipitated chromatin (%IP). Error bars indicate S.D. from 3 biological replicates. Mean values marked with different letter (a or b) indicate results significantly different from each other, as established by One Way ANOVA and Holm-Sidak test for multiple comparison (P<0.01).
