# Supplementary material for: Telomeric Repeats Facilitate CENP-ACnp1 Incorporation via Telomere Binding Proteins
Source: PLoS One. 2013 Jul 31;8(7):e69673. doi: 10.1371/journal.pone.0069673 (PMC3729655; doi:10.1371/journal.pone.0069673)
Supplement: Figure S7 — (A) ChIP-qPCR of CENP-ACnp1 levels at TM1 in the central region of centromere 1 and centromeric otr dgI repeats in wild-type (wt) and mutant cells (ccq1∆, taz1∆, and clr4∆) which contain the internal telomere (ura4 +: Int-Telo) and that express endogenous (Endog. CENP-ACnp1) or additional (nmt41-CENP-ACnp1) levels of CENP-ACnp1. (B) ChIP-qPCR of H3K9me2 levels in the same cell backgrounds. Enrichment at TM1 is reported as the percentage of immunoprecipitated chromatin (% IP). Enrichment on dgI was normalized the background signal obtained for the gene encoding actin (act1 +). Error bars indicate S.D. from 3 biological replicates. Mean values marked with different letter (a, b or c) indicate results significantly different from each other, as established by One Way ANOVA and Holm-Sidak test for multiple comparison (P<0.01). (PDF) [file pone.0069673.s007.pdf]

Figure S7

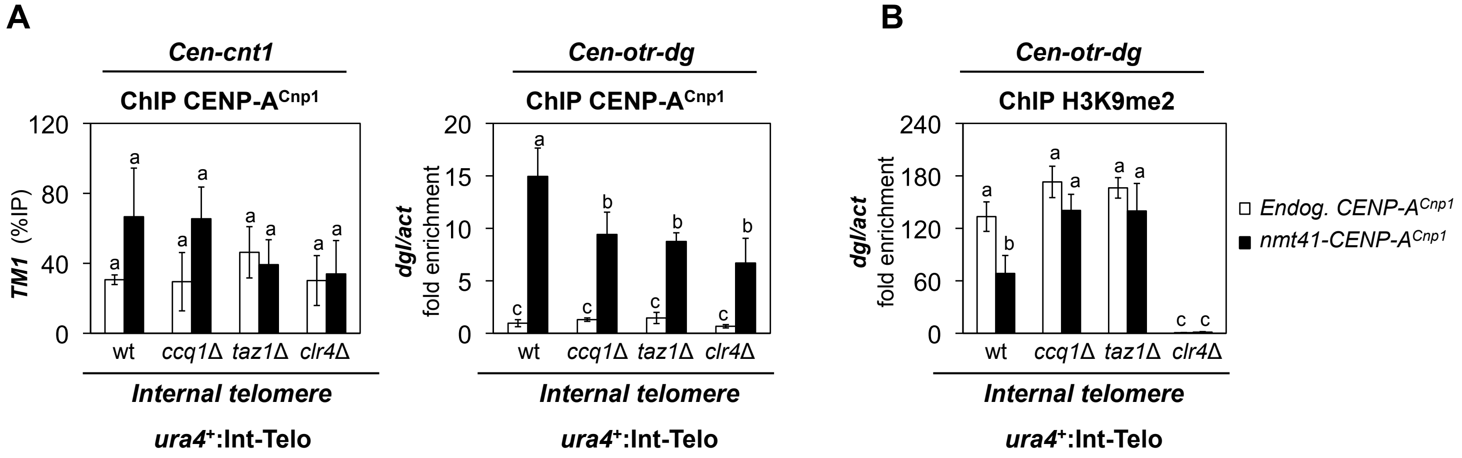

Figure S7. Enrichment of CENP-A^{Cnp1} and H3K9me2 at centromeres in wild-type cells and mutants that affect telomeric chromatin

(A) ChIP-qPCR of CENP-A^{Cnp1} levels at TM1 in the central region of centromere 1 and centromeric otr *dgI* repeats in wild-type (wt) and mutant cells (*ccq1*Δ, *taz1*Δ, and *clr4*Δ) which contain the internal telomere (*ura4*$^{+}$:Int-Telo) and that express endogenous (Endog. CENP-A^{Cnp1}) or additional (*nmt41-CENP-A*^{Cnp1}) levels of CENP-A^{Cnp1} **(B)** ChIP-qPCR of H3K9me2 levels in the same cell backgrounds. Enrichment at *TM1* is reported as the percentage of immunoprecipitated chromatin (%IP). Enrichment on *dgI* was normalized the background signal obtained for the gene encoding actin (*act1+*). Error bars indicate S.D. from 3 biological replicates. Mean values marked with different letter (a, b or c) indicate results significantly different from each other, as established by One Way ANOVA and Holm-Sidak test for multiple comparison (P<0.01).
