# Supplementary material for: Telomeric Repeats Facilitate CENP-ACnp1 Incorporation via Telomere Binding Proteins
Source: PLoS One. 2013 Jul 31;8(7):e69673. doi: 10.1371/journal.pone.0069673 (PMC3729655; doi:10.1371/journal.pone.0069673)
Supplement: Table S1 — (PDF) [file pone.0069673.s008.pdf]

Table S1. List of strains used in this study

Strain	***Genotype***	***Reference***
520	*h+ leul-32 ade6-210 ura4-DS/E [Ch16 ade6-216 m23::ura4-Tel (pEN72)]*	*Nimmo et al., 1994*
566	*h+ leul-32 ade6-210 ura4-DS/E [Ch16 ade6-216 m23::ura4-TAS-Tel (pEN76)]*	*Nimmo et al., 1994*
4133	*h+ ade6-210 leu1-32 arg3-D4 his3-D1 ura4-DS/E*	
4134	*h- ade6-210 leu1-32 arg3-D4 his3-D1 ura4-DS/E*	
10325	*h- ars1(MluI)::pREP3X-leu2Sc leu1-32*	
10328	*h- ars1(MluI)::pREP81XCnp1-leu2Sc leu1-32*	
10332	*h- ars1(MluI)::pREP41XCnp1-leu2Sc leu1-32*	
10336	*h- ars1(MluI)::pREP3XCnp1-leu2Sc leu1-32*	
11819	*h- ars1(MluI)::pREP3X-leu2Sc [Ch16 ade6-216 m23::ura4-TAS-Tel (pEN76)] ade6-210 leu1-32 his3-D1 ura4-DS/E*	
11824	*h- ars1(MluI)::pREP41XCnp1-leu2Sc [Ch16 ade6-216 m23::ura4-TAS-Tel (pEN76)] ade6-210 leu1-32 ura4-DS/E*	
11825	*h- ars1(MluI)::pREP3X-leu2Sc [Ch16 ade6-216 m23::ura4-Tel (pEN72)] ade6-210 leu1-32 his3-D1 ura4-DS/E*	
11829	*h- ars1(MluI)::pREP41XCnp1-leu2Sc [Ch16 ade6-216 m23::ura4-Tel (pEN72)] ade6-210 leu1-32 his3-D1 ura4-DS/E*	
13238	*h- clr4Δ ::nat ars1(MluI)::pREP3X-leu2Sc [Ch16 ade6-216 m23::ura4-Tel (pEN72)] ade6-210 ura4D-S/E leu1-32 **	
13243	*h- clr4Δ::nat ars1(MluI)::pREP41XCnp1-leu2Sc [Ch16 ade6-216 m23::ura4-Tel (pEN72)] ade6-210 ura4D-S/E leu1-32 **	
13255	*h- clr3Δ::kanMX6 ars1(MluI)::pREP3X-leu2Sc [Ch16 ade6-216 m23::ura4-Tel (pEN72)] ade6-210 ura4D-S/E leu1-32 **	
13260	*h- clr3Δ::kanMX6 ars1(MluI)::pREP41XCnp1-leu2Sc [Ch16 ade6-216 m23::ura4-Tel (pEN72)] ade6-210 ura4D-S/E leu1-32 **	
18403	*h+ taz1Δ::kanMX6*	*From J.P. Cooper*
18406	*h- ccq1Δ::hygMX6*	*Tomita&Cooper. 2008*
18405	*h+ ura4:telo ade6-210 his3-D1 leu1-32*	*Miller et al 2006*
18500	*h+ rif1Δ::hygMX6 ade6-216 leu1-32 ura4-D18 his3-D1*	*From J.P. Cooper*
18559	*h- taz1Δ::kanMX6 ars1(MluI)::pREP3X-leu2Sc [Ch16 ade6-216 m23::ura4-Tel (pEN72)] ade6-210 leu1-32 ura4-DS/E **	
18563	*h- taz1Δ::kanMX6 ars1(MluI)::pREP41XCnp1-leu2Sc [Ch16 ade6-216 m23::ura4-Tel (pEN72)] leu1-32 ade6-210 ura4-DS/E **	
18567	*h- ccq1Δ::hygMX6 ars1(MluI)::pREP3X-leu2Sc [Ch16 ade6-216 m23::ura4-Tel (pEN72)] leu1-32 ade6-210 ura4-DS/E **	
18569	*h- ccq1Δ::hygMX6 ars1(MluI)::pREP41XCnp1-leu2Sc [Ch16 ade6-216 m23::ura4-Tel (pEN72)] leu1-32 ade6-210 ura4-DS/E **	
18571	*h- rif1Δ::hygMX6 ars1(MluI)::pREP3X-leu2Sc [Ch16 ade6-216 m23::ura4-Tel (pEN72)] leu1-32 ade6-210 ura4-DS/E his3-D1*	
18574	*h- rif1Δ::hygMX6 ars1(MluI)::pREP41XCnp1-leu2Sc [Ch16 ade6-216 m23::ura4-Tel (pEN72)] leu1-32 ade6-210 ura4-DS/E his3-D1*	
18596	*h- ura4:telo ars1(MluI)::pREP3X-leu2Sc leu1-32 ade6-210 **	
18605	*h- ura4:telo ars1(MluI)::pREP41XCnp1-leu2Sc leu1-32 **	
18663	*h- ura4:telo taz1Δ::kanMX6 ars1(MluI)::pREP3X-leu2Sc leu1-32 ade6-210 **	
18664	*h- ura4:telo taz1Δ::kanMX6 ars1(MluI)::pREP41XCnp1-leu2Sc leu1-32 **	
18671	*h- ura4:telo ccq1Δ::hygMX6 ars1(MluI)::pREP3X-leu2Sc leu1-32 ade6-210 **	
18675	*h- ura4:telo ccq1Δ::hygMX6 ars1(MluI)::pREP41XCnp1-leu2Sc leu1-32 **	
18685	*h- ura4:telo clr4Δ::nat ars1(MluI)::pREP3X-leu2Sc leu1-32 ade6-210 **	
18687	*h- ura4:telo clr4Δ::nat ars1(MluI)::pREP41XCnp1-leu2Sc leu1-32 **	

* only relevant genotype is shown
