# Supplementary material for: Telomeric Repeats Facilitate CENP-ACnp1 Incorporation via Telomere Binding Proteins
Source: PLoS One. 2013 Jul 31;8(7):e69673. doi: 10.1371/journal.pone.0069673 (PMC3729655; doi:10.1371/journal.pone.0069673)
Supplement: Table S2 — (PDF) [file pone.0069673.s009.pdf]

Table S2. Primers used for qPCR

Primer name	***Locus***	***Sequence (5´-3´)***
q-cnt1-F	*TM1*	CAGACAATCGCATGGTACTATC
q-cnt1-R	*TM1*	AGGTGAAGCGTAAGTGAGTG
q-dgI-F	*dgI*	AATTGTGGTGGTGTGGTAATAC
q-dgI-R	*dgI*	GGGTTCATCGTTTCCATTCAG
q-act1-f	*act1*$^{+}$	CCCAAATCCAACCGTGAGAAGATG
q-act1-r	*act1*$^{+}$	CCAGAGTCCAAGACGATACCAGTG
q-ura4-f2	*ura4*$^{+}$	AGGCTCTTTGGCTACTGGTTCC
q-ura4-r2	*ura4*$^{+}$	AGTTATGTAGTCGCTTTGAAGGTTAGG
q-tel1R-f	Ch I, right arm, subtelomeric (53 kb)	AACGATGAACCTTTTTCTTATCCG
q-tel1R-r	Ch I, right arm, subtelomeric (53 kb)	AAAGGATTAACGCCTTTCCTCG
