# Supplementary material for: Telomeric Repeats Facilitate CENP-ACnp1 Incorporation via Telomere Binding Proteins
Source: PLoS One. 2013 Jul 31;8(7):e69673. doi: 10.1371/journal.pone.0069673 (PMC3729655; doi:10.1371/journal.pone.0069673)
Supplement: Table S3 — (PDF) [file pone.0069673.s010.pdf]

Table S3. Primers used for multiplex PCR

Primer name	*Location*	*Distance from distal end*	*Sequence (5´-3´)*
AGC34	Ch I, right arm	21.7 kb	TTCTACAATTAGCTGAACCC
AGC35	Ch I, right arm	21.7 kb	TAATGGCTTCAATAATTGGC
AGC32	Ch I, right arm	53 kb	ATGAACCTTTTTCTTATCCG
AGC33	Ch I, right arm	53 kb	CGGTATTTGTTTTTAAGTACG
AGC38	Ch I, right arm	72 kb	CATTAAGAAAAACCACTACAG
AGC39	Ch I, right arm	72 kb	TGCTGAACTTCATAAAATCGG
AGC60	Ch II, right arm	3.7 kb	TTAAAAAAAGTTAAGGGTAGG
AGC61	Ch II, right arm	3.7 kb	CTTTTTCTCTATTTGTTTCATC
AGC40	Ch II, right arm	47.9 kb	CCCTTAATTTAAATACGAGGC
AGC41	Ch II, right arm	47.9 kb	CAGTATTAAATATGCAAGCGC
AGC46	Ch III, left arm	34.5 kb	CACATGGAAATGAAAATAAGCG
AGC47	Ch III, left arm	34.5 kb	AACATCTTCACAAACTGCTACC
act-for	*act1*$^{+}$	-----	GGCATCACACTTTCTACAACG
act-rev	*act1*$^{+}$	-----	GAGTCCAAGACGATACCAGTG
